# Analysis of the immune-inflammatory indices for patients with metastatic hormone-sensitive and castration-resistant prostate cancer

**DOI:** 10.1186/s12885-024-12593-z

**Published:** 2024-07-09

**Authors:** Zhipeng Wang, Haoyang Liu, Qiyu Zhu, Junru Chen, Jinge Zhao, Hao Zeng

**Affiliations:** 1 Department of Urology, Institute of Urology, West China Hospital, Sichuan University, Guoxue Alley, No. 37, Chengdu, Sichuan 610041 People’s Republic of China; 2 Department of Urology, Sichuan Provincial People’s Hospital, University of Electronic Science and Technology of China, Chengdu, Sichuan 610031 People’s Republic of China

**Keywords:** Immune-inflammatory Indices, Metastatic Hormone-sensitive Prostate Cancer, Metastatic Castration-resistant Prostate Cancer, Biomarker, Cancer Prognosis

## Abstract

**Background:**

Inflammation plays a pivotal role in the progression of prostate cancer (PCa). Several immune-inflammatory indices, including neutrophil to lymphocyte ratio (NLR), derived neutrophil to lymphocyte ratio (dNLR), lymphocyte to monocyte ratio (LMR) and platelet to lymphocyte ratio (PLR), lung immune prognostic index (LIPI), systemic inflammation response index (SIRI) and systemic immune inflammation index (SII), have demonstrated their prognostic values in several solid malignancies. However, Comparisons of superiority with these seven indices’ predictive efficacy within metastatic hormone-sensitive PCa (mHSPC) and metastatic castration-resistant PCa (mCRPC) remain uncertain.

**Methods:**

We retrospectively included 407 patients diagnosed with mHSPC and 158 patients with mCRPC at West China Hospital from 2005 to 2022. The seven immune-inflammatory indices were computed based on hematological data of mHSPC at initial diagnosis and mCRPC at progression to CRPC. Prognostic value for castration-resistant prostate cancer-free survival (CFS), overall survival (OS), prostate-specific antigen progression-free survival (PSA-PFS) and prostate-specific antigen (PSA) response was assessed using Kaplan–Meier curves, Cox regression models, and chi-square tests. The predictive performance of each immune-inflammatory index was assessed using the area under the curve (AUC) in time-dependent receiver operating characteristic curve (ROC) analysis and C-index calculation.

**Results:**

All seven immune-inflammatory indices were significantly associated with CFS and OS in the mHSPC cohort, as well as with PSA response, PSA-PFS, and OS in the mCRPC cohort. In the mHSPC cohort, LIPI consistently exhibited higher AUC values compared to NLR, dNLR, LMR, PLR, SII, and SIRI for predicting CFS and OS. This indicates that LIPI had a superior discriminative ability compared to the other indices (C-index of LIPI: 0.643 and 0.686 for CFS and OS, respectively). Notably, the predictive advantage of LIPI over other indices in the mHSPC stage diminished in the mCRPC stage.

**Conclusions:**

This study firstly confirmed the prognostic value of SII, SIRI and LIPI in mHSPC and mCRPC, and revealed that LIPI had a higher predictive power than NLR, dNLR, LMR, PLR, SII and SIRI in mHSPC. These non-invasive indices can enable clinicians to quickly assess the prognosis of patients.

**Supplementary Information:**

The online version contains supplementary material available at 10.1186/s12885-024-12593-z.

## Background

Prostate cancer (PCa) is the most common and the second deadliest cancer among men worldwide, with 6% to 44% of cases being metastatic prostate cancer (mPCa) at diagnosis [1–3]. Despite advancements in treatment, patients inevitably progress from metastatic hormone-sensitive prostate cancer (mHSPC) to the terminal stage of metastatic castration-resistant prostate cancer (mCRPC). Inflammation is a key factor that drives PCa malignancy [4, 5]. It can induce DNA damage, activate oncogenic pathways, and generate “inflammatory storms” that promote mutation and immune evasion, leading to PCa progression [4].

Previous studies have shown the prognostic value of inflammatory biomarkers in mPCa, such as neutrophil to lymphocyte ratio (NLR), derived neutrophil to lymphocyte ratio (dNLR), lymphocyte to monocyte ratio (LMR) and platelet to lymphocyte ratio (PLR) [6–8]. However, most studies focused on the terminal stage of mCRPC, and there is a lack of research on newly diagnosed mHSPC. Moreover, some novel hematological immune-inflammatory indices have emerged, such as lung immune prognostic index (LIPI), systemic inflammation response index (SIRI) and systemic immune inflammation index (SII). LIPI is a biomarker based on dNLR and lactate dehydrogenase (LDH), which was initially developed for lung cancer immunotherapy and then proved to have predictive value beyond immunotherapy or lung cancer [9–12]. Based on neutrophils, platelets, and lymphocytes, SII was first reported to predict the prognosis of hepatocellular carcinoma patients, and later shown to be more accurate than NLR and PLR for colorectal and esophageal cancer patients [13–15]. Based on monocytes, neutrophils and lymphocytes counts, SIRI was first reported to predict the survival of pancreatic cancer patients, and subsequently proven to predict the prognosis of various tumors, such as breast, esophageal, and cervical cancer [16–19]. Although LIPI, SII and SIRI have been proven to have predictive value in various types of tumors, their applicability in mHSPC and mCRPC remains unclear.

Therefore, the aim of this study was to verify the applicability of the latest immune- inflammatory indices in mHSPC and mCRPC and compare the predictive power of the novel and traditional immune-inflammatory indices.

## Methods

### Study population and design

We retrospectively enrolled 407 patients with newly diagnosed mHSPC and 158 patients with mCRPC at West China Hospital from 2005 to 2022. All newly diagnosed mHSPC patients received maximal androgen blockade therapy, which consisted of surgical castration or medical castration (goserelin 3.6 mg/month or 10.8 mg/3 months) plus bicalutamide 50 mg/day. All the mCRPC patients received castration therapy (goserelin 3.6 mg/month or 10.8 mg/3 months) plus abiraterone (1000 mg/day plus prednisone 10 mg/day) as the first-line treatment. Hematological data, including white blood cell (WBC) count, platelet count (PLT), lactate dehydrogenase (LDH) level, absolute neutrophil count (ANC), absolute lymphocyte count (ALC), and absolute monocyte count (AMC), were retrospectively collected for mHSPC patients at initial diagnosis and mCRPC patients at progression to CRPC. These data were used to calculate the seven immune-inflammatory indices: NLR (ANC/ALC), dNLR (ANC/(WBC-ANC)), LMR (ALC/AMC), PLR (PLT/ALC), SII (PLT × ANC/ALC), SIRI (AMC × ANC/ALC), and LIPI (based on dNLR and LDH level). When calculating LIPI, values exceeding 3.0 for dNLR and surpassing the upper limit of normal for LDH were considered independent risk factors, categorizing patients into three groups: 0 risk factors denoted LIPI-good, 1 risk factor indicated LIPI-intermediate, and 2 risk factors signified LIPI-poor. In addition, further baseline clinicopathological data were collected, encompassing age, prostate-specific antigen (PSA), Gleason score (GS), Eastern Cooperative Oncology Group (ECOG) score, visceral metastasis status, hemoglobin (HGB) levels, and alkaline phosphatase (ALP) levels. It should be noted that to mitigate the interference of infectious diseases on the predictive efficacy of the immune-inflammatory indices, mPCa patients diagnosed with infectious diseases were excluded.

### Endpoints

The endpoints for mHSPC patients included castration-resistant prostate cancer-free survival (CFS) and overall survival (OS). CFS was defined as the time from diagnosis to progression to CRPC with reference to the EAU guidelines in 2022 [20]. OS for mHSPC patients was defined as the time from diagnosis to death. The endpoints for mCRPC patients included PSA response after ABI treatment, prostate-specific antigen progression-free survival (PSA-PFS) and OS. PSA response was defined as a decrease of more than or equaling to 50% in PSA level after ABI treatment and maintained for at least 4 weeks. PSA-PFS was defined as the duration from starting ABI treatment to PSA progression. And PSA progression was defined as a consecutive increase in PSA level twice after starting treatment, with an increase of 25% or more from the baseline, and an absolute value exceeding 2 ng/ml. OS for mCRPC patients was defined as the time from starting ABI treatment to death.

### Statistical analysis

CFS, PSA-PFS and OS were assessed by the Kaplan–Meier curves and compared using the log-rank analysis. Chi-square test was used to analyze the differences in PSA response among different groups. The Cox regression model was used to evaluate the clinicopathological factors for CFS, PSA-PFS, and OS via the univariate and multivariate analyses. The predictive performance of each immune-inflammatory index was assessed using the area under the curve (AUC) in time-dependent receiver operating characteristic curve (ROC) analysis and C-index calculation. All statistical analyses were performed using R version 4.1.0 and a *P* value < 0.05 was considered statistically significant.

## Results

### Patient characteristics

The baseline characteristics of 407 mHSPC patients and 158 mCRPC patients are summarized in Tables 1 and 2. The median follow-up time of mHSPC patients was 60.8 months, during which 323 (79.4%) mHSPC patients progressed to mCRPC and 184 (45.2%) patients died. The median CFS and median OS of mHSPC patients were 16.6 months and 69.5 months, respectively. The median follow-up time of mCRPC patients was 49.7 months, during which 138/158 (87.3%) mCRPC patients had PSA progression after first-line ABI treatment, 51/158 (32.3%) patients received sequential treatment following ABI failure (see Table S1 for details), and 94 (59.5%) patients died. The median PSA-PFS and median OS of mCRPC patients were 9.6 months and 38.0 months, respectively.

**Table 1:** Baseline characteristics of the patients with mHSPC

mHSPC cohort (*N* = 407)	
**Age (years)**	
Median value (range)	72.0 (44.0–90.0)
**ECOG score**	
0–1, N (%)	351 (86.2)
≥ 2, N (%)	56 (13.8)
**ISUP group**	
1–3, N (%)	65 (16.0)
4, N (%)	76 (18.7)
5, N (%)	266 (65.4)
**Visceral metastases**	
Yes, N (%)	46 (11.3)
No, N (%)	361 (88.7)
**PSA (ng/ml)**	
Median value (range)	100.1 (4.1–5200.0)
**Therapy information**	
First-line Therapy	MAB
**HGB (g/L)**	
Median value (range)	131.0 (54.0–181.0)
**ALP (IU/L)**	
Median value (range)	104 (36.0–5692.0)
**LDH (U/L)**	
Median value (range)	202.0 (88.0–3740.0)
**NLR**	
Median value (range)	2.8 (0.0–29.6)
**dNLR**	
Median value (range)	1.9 (0.2–17.5)
**LMR**	
Median value (range)	3.4 (0.4–2841.7)
**PLR**	
Median value (range)	121.4 (0.1–686.2)
**SII**	
Median value (range)	469.7 (0.7–6634.4)
**SIRI**	
Median value (range)	1.2 (0.0–30.7)
**LIPI**	
LIPI-Good, N (%)	206 (50.6)
LIPI-Inter., N (%)	142 (34.9)
LIPI-Poor, N (%)	59 (14.5)
**CFS (months)**	
Median value (range)	16.6 (14.8–18.4)
**OS (months)**	
Median value (range)	69.5 (57.8–83.1)

*mHSPC* metastatic hormone-sensitive prostate cancer, *ECOG* Eastern Cooperative Oncology Group, *ISUP* International Society of Urological Pathology, *PSA* Prostate-specific antigen, *MAB* Maximum androgen blockage, *HGB* Hemoglobin, *ALP* Alkaline phosphatase, *LDH* Lactate dehydrogenase, *NLR* Neutrophil to lymphocyte ratio, *dNLR* Derived neutrophil to lymphocyte ratio, *LMR* Lymphocyte to monocyte ratio, *PLR* Platelet to lymphocyte ratio, *SII* Systemic immune inflammation index, *SIRI* Systemic inflammation response index, *LIPI* Lung immune prognostic index, *CFS* castration-resistant prostate cancer-free survival, *OS* Overall survival

**Table 2:** Baseline characteristics of the patients with mCRPC

mCRPC cohort (*N* = 158)	
**Age (years)**	
Median value (range)	71.0 (51.0–91.0)
**ECOG score**	
0–1, N (%)	146 (92.4)
≥ 2, N (%)	12 (7.6)
**CFS (months)**	
Median value (range)	12.5 (1.3–97.5)
**ISUP group**	
1–3, N (%)	17 (10.8)
4, N (%)	35 (22.2)
5, N (%)	106 (67.1)
**Visceral metastases**	
Yes, N (%)	20 (12.7)
No, N (%)	138 (87.3)
**PSA (ng/ml)**	
Median value (range)	12.3 (1.2–7796.0)
**Therapy information**	
First-line therapy	Abiraterone
Number of patients received sequential treatment	51 (32.3%)
≥ 3 Therapies	17 (10.8%)
**HGB (g/L)**	
Median value (range)	128.0 (68.0–156.0)
**ALP (IU/L)**	
Median value (range)	103.5 (34.0–2954.0)
**LDH (U/L)**	
Median value (range)	218.5 (99.0–2364.0)
**NLR**	
Median value (range)	2.4 (1.0–29.0)
**dNLR**	
Median value (range)	1.7 (0.6–7.4)
**LMR**	
Median value (range)	3.5 (0.6–17.7)
**PLR**	
Median value (range)	111.7 (23.8–444.7)
**SII**	
Median value (range)	442.7 (88.5–2973.5)
**SIRI**	
Median value (range)	1.2 (0.3–15.6)
**LIPI**	
LIPI-Good, N (%)	70 (44.3)
LIPI-Inter., N (%)	69 (43.7)
LIPI-Poor, N (%)	19 (12.0)
**PSA response (PSA 50)**	
No, N (%)	62 (39.2)
Yes, N (%)	96 (60.8)
**PSA-PFS (months)**	
Median value (range)	9.6 (8.1–12.3)
**OS (months)**	
Median value (range)	38.0 (32.3–53.4)

*mCRPC* metastatic castration-resistant prostate cancer, *ECOG* Eastern Cooperative Oncology Group, *ISUP* International Society of Urological Pathology, *PSA* Prostate-specific antigen, *HGB* Hemoglobin, *ALP* Alkaline phosphatase, *LDH* Lactate dehydrogenase, *NLR* Neutrophil to lymphocyte ratio, *dNLR* Derived neutrophil to lymphocyte ratio, *LMR* Lymphocyte to monocyte ratio, *PLR* Platelet to lymphocyte ratio, *SII* Systemic immune inflammation index, *SIRI* Systemic inflammation response index, *LIPI* Lung Immune Prognostic Index, *CFS* Castration-resistant prostate cancer-free survival, *PSA-PFS* Prostate-specific antigen progression-free survival, *OS* Overall survival

### The analysis of optimal cut-off points

**Fig. 1:**
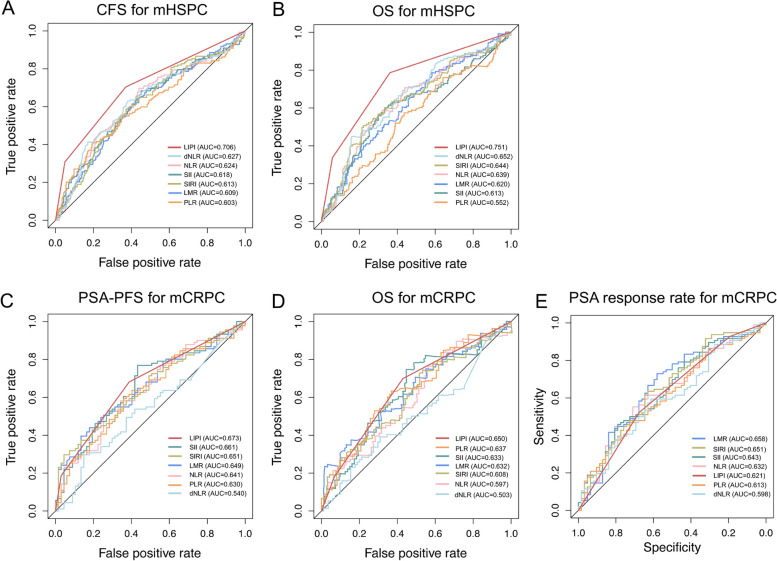
The predictive performance of NLR, dNLR, LMR, PLR, SII, SIRI and LIPI in mHSPC and mCRPC cohorts. ROC analyses and AUC values of the seven indices: **A** CFS for mHSPC cohort, **B** OS for mHSPC cohort, **C** PSA-PFS for mCRPC cohort, **D** OS for mCRPC cohort, and (**E**) PSA response rate for mCRPC cohort. NLR: neutrophil to lymphocyte ratio; dNLR: derived neutrophil to lymphocyte ratio; LMR: lymphocyte to monocyte ratio; PLR: platelet to lymphocyte ratio; SII: systemic immune inflammation index; SIRI: systemic inflammation response index; LIPI: lung immune prognostic index; mHSPC: metastatic hormone-sensitive prostate cancer; mCRPC: metastatic castration-resistant prostate cancer; ROC: receiver operating characteristic curve; AUC: area under the curve; CFS: castration-resistant prostate cancer-free survival; OS: overall survival; PSA: prostate-specific antigen; PSA-PFS: prostate-specific antigen progression-free survival

For the six immune-inflammatory indices, including NLR, dNLR, LMR, PLR, SIRI, and SII, with the exception of LIPI, there is no consensus on the optimal cut-off values. Consequently, we performed ROC curve analysis to determine the optimal cut-off points based on the principle of maximizing the Youden index (Fig. 1). The optimal cut-off points for NLR, dNLR, LMR, PLR, SIRI, and SII are listed in Table S2.

### Predictive value of immune-inflammatory indices

#### Predictive value of the novel immune-inflammatory indices—SII, SIRI, and LIPI

**Fig. 2:**
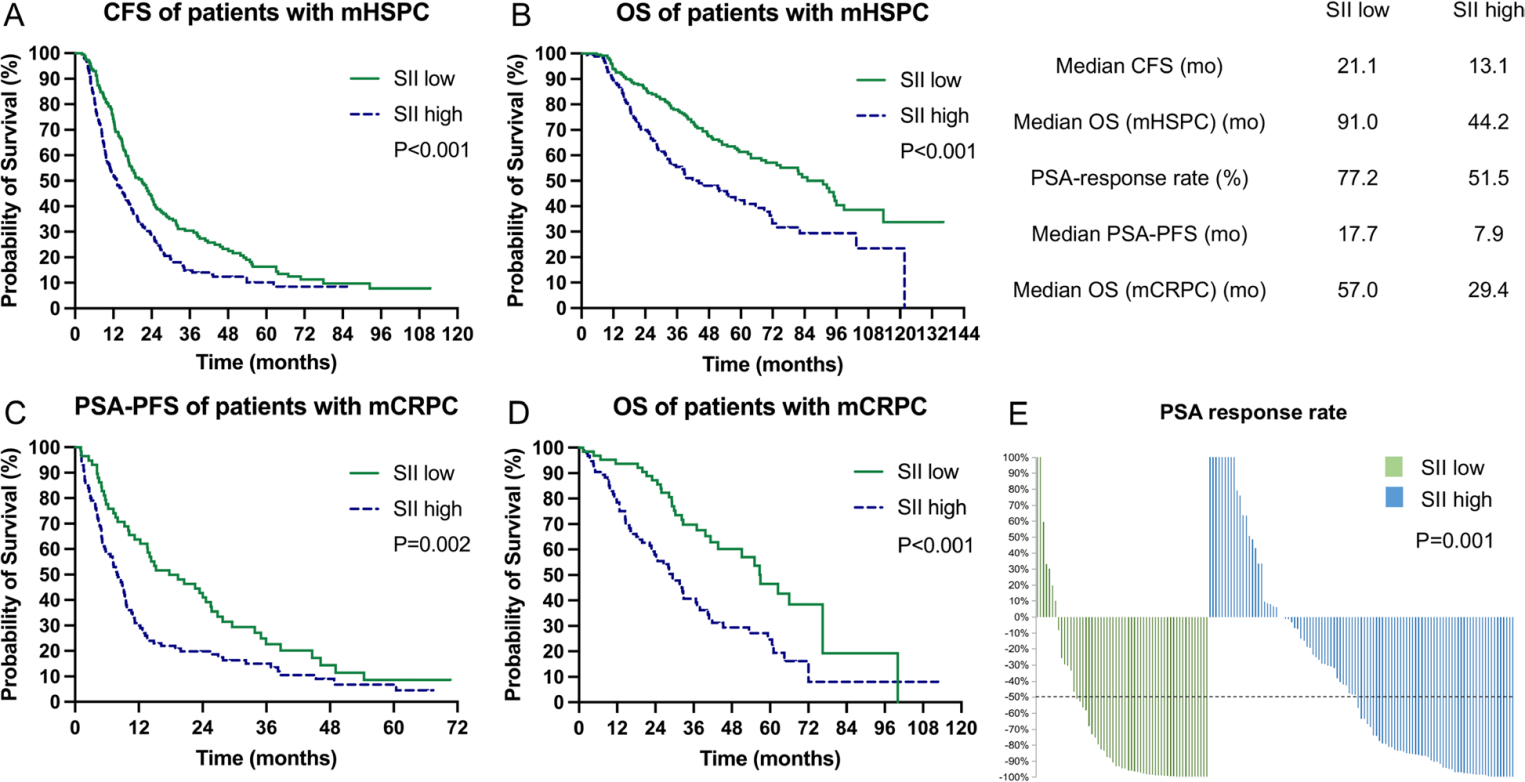
The prognostic value of SII in mHSPC and mCRPC cohorts. **A** Kaplan–Meier curves of CFS for mHSPC cohort; **B** Kaplan–Meier curves of OS for mHSPC cohort; **C** Kaplan–Meier curves of PSA-PFS for mCRPC cohort; **D** Kaplan–Meier curves of OS for mCRPC cohort; **E** PSA response rate for mCRPC cohort. SII: systemic immune inflammation index; mHSPC: metastatic hormone-sensitive prostate cancer; mCRPC: metastatic castration-resistant prostate cancer; CFS: castration-resistant prostate cancer-free survival; OS: overall survival; PSA: prostate-specific antigen; PSA-PFS: prostate-specific antigen progression-free survival

**Fig. 3:**
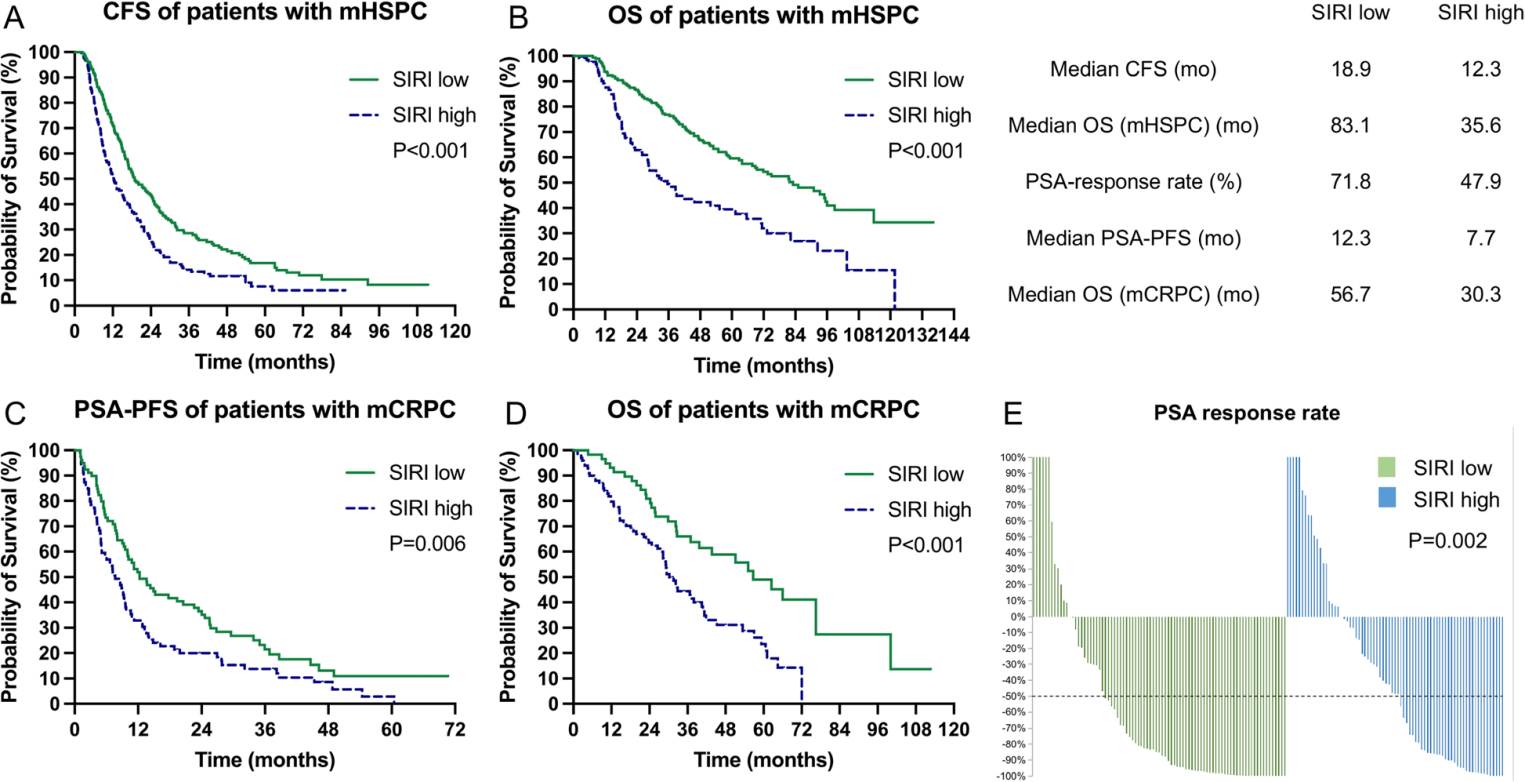
The prognostic value of SIRI in mHSPC and mCRPC cohorts. **A** Kaplan–Meier curves of CFS for mHSPC cohort; **B** Kaplan–Meier curves of OS for mHSPC cohort; **C** Kaplan–Meier curves of PSA-PFS for mCRPC cohort; **D** Kaplan–Meier curves of OS for mCRPC cohort; **E** PSA response rate for mCRPC cohort. SIRI: systemic inflammation response index; mHSPC: metastatic hormone-sensitive prostate cancer; mCRPC: metastatic castration-resistant prostate cancer; CFS: castration-resistant prostate cancer -free survival; OS: overall survival; PSA: prostate-specific antigen; PSA-PFS: prostate-specific antigen progression-free survival

**Fig. 4:**
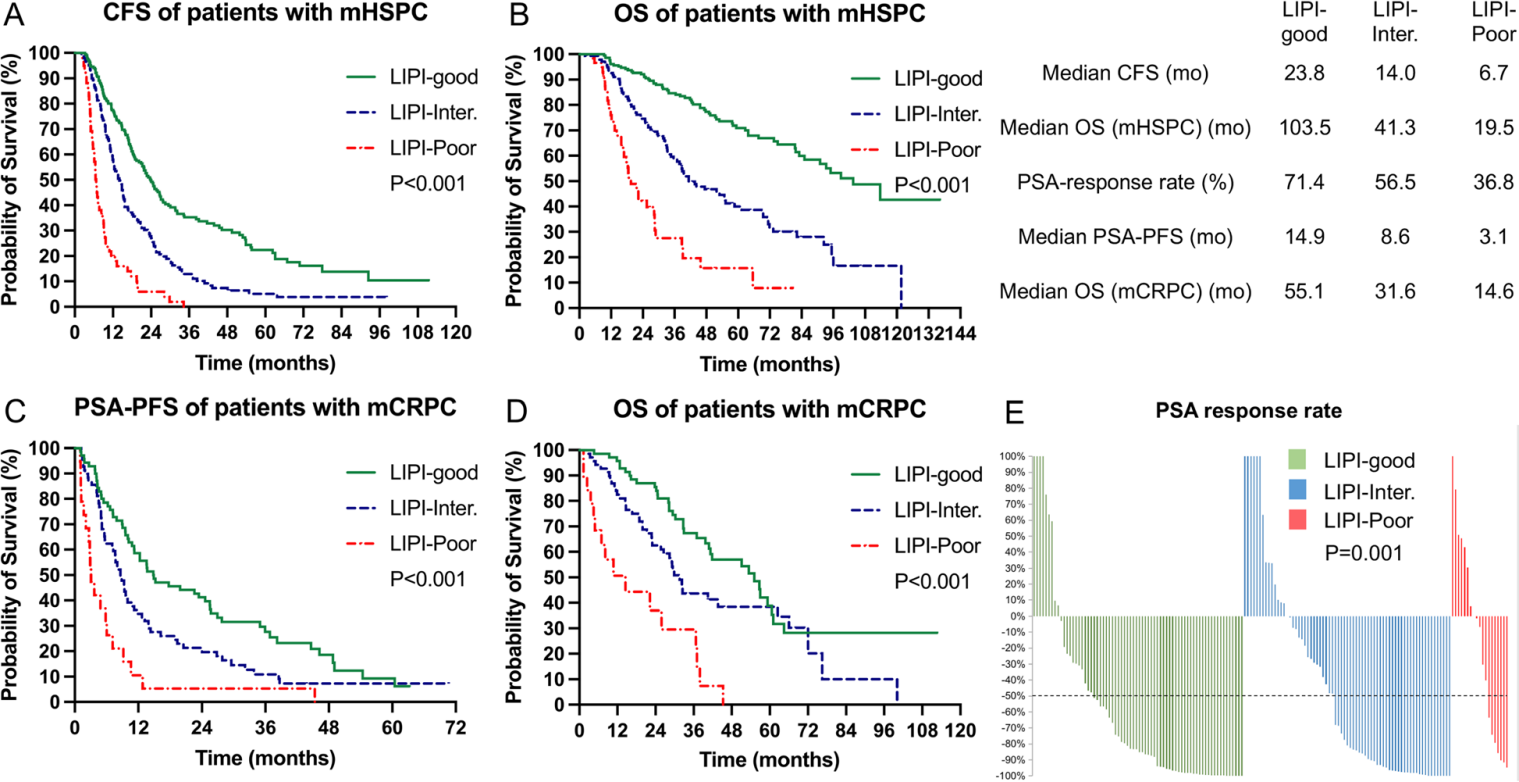
The prognostic value of LIPI in mHSPC and mCRPC cohorts. **A** Kaplan–Meier curves of CFS for mHSPC cohort; **B** Kaplan–Meier curves of OS for mHSPC cohort; **C** Kaplan–Meier curves of PSA-PFS for mCRPC cohort; **D** Kaplan–Meier curves of OS for mCRPC cohort; **E** PSA response rate for mCRPC cohort. LIPI: lung immune prognostic index; mHSPC: metastatic hormone-sensitive prostate cancer; mCRPC: metastatic castration-resistant prostate cancer; CFS: castration-resistant prostate cancer-free survival; OS: overall survival; PSA: prostate-specific antigen; PSA-PFS: prostate-specific antigen progression-free survival

We confirmed the applicability of the novel immune-inflammatory indices SII, SIRI, and LIPI in both mHSPC and mCRPC. In mHSPC cohort, patients with higher SII and SIRI had a significantly reduced CFS (mCFS for SII: 13.1 mo vs. 21.1 mo, *P* < 0.001; mCFS for SIRI: 12.3 mo vs. 18.9 mo, *P* < 0.001) and OS (mOS for SII: 44.2 mo vs. 91.0 mo, *P* < 0.001; mOS for SIRI: 35.6 mo vs. 83.1 mo, *P* < 0.001) compared to those with lower SII (Fig. 2A and B) and SIRI (Fig. 3A and B), respectively. Stratifying mHSPC patients into LIPI-good (mCFS: 23.8 mo; mOS: 103.5 mo), LIPI-inter (mCFS: 14.0 mo; mOS: 41.3 mo), and LIPI-poor groups (mCFS: 6.7 mo; mOS: 19.5 mo) revealed sequentially worse outcomes (*P* < 0.001 for all) (Fig. 4A and B). Cox regression confirmed that SII, SIRI, and LIPI independently prognosticated CFS and OS in mHSPC cohort (Table S3 and Table S4).

In mCRPC cohort, patients with higher SII and SIRI had lower PSA response rate (SII-PSA response rate 51.5% [52/101] vs. 77.2% [44/57], *P* = 0.001; SIRI-PSA response rate 47.9% [35/73] vs. 71.8% [61/85], *P* = 0.002;), expedited PSA progression (SII-PSA-PFS: 7.9 mo vs. 17.7 mo, *P* = 0.002; SIRI-PSA-PFS: 7.7 mo vs. 12.3 mo, *P* = 0.006) and inferior OS (SII-mOS: 29.4 mo vs. 57.0 mo, *P* < 0.001; SIRI-OS: 30.3 mo vs. 56.7 mo, *P* < 0.001) than those with lower indices, respectively (Figs. 2C to E and 3C to E). mCRPC patients in the LIPI-good, LIPI-inter, and LIPI-poor groups showed a ladder-shaped worse PSA response (71.4% [50/70] vs. 56.5% [39/69] vs. 36.8% [7/19]; *P* < 0.001), PSA-PFS (14.9 mo vs. 8.6 mo vs. 3.1 mo; *P* < 0.001), and OS (55.1 mo vs. 31.6 mo vs. 14.6 mo; *P* < 0.001) (Fig. 4C to E). Cox regression further confirmed the independent prognostic significance of SII, SIRI, and LIPI for PSA-PFS and OS in mCRPC cohort (Table S5 and Table S6).

Given that cut-off values for the novel immune-inflammatory indices SII and SIRI has yet to be established, we conducted supplementary Cox regression, treating SII and SIRI as continuous variables in mHSPC and mCRPC patients, to further validated their predictive efficacy (Table S7, S8, S9, and S10).

#### Novel and traditional immune-inflammatory indices: comparison of predictive power

Before comparing the predictive power of novel and traditional immune-inflammatory indices, we first validated the prognostic value of traditional inflammatory parameters NLR, dNLR, LMR, and PLR. In mHSPC patients. Those with higher NLR, dNLR and PLR had significantly shorter CFS (mCFS for NLR: 12.3 mo vs. 21.3 mo, *P* < 0.001; mCFS for dNLR: 12.1 mo vs. 18.9 mo, *P* < 0.001; mCFS for PLR: 11.0 mo vs. 18.4 mo, *P* < 0.001) and OS (mOS for NLR: 39.1 mo vs. 83.1 mo, *P* < 0.001; mOS for dNLR: 45.8 mo vs. 91.0 mo, *P* < 0.001; mOS for PLR: 58.0 mo vs. 81.5 mo, *P* = 0.016) than those with lower indices, respectively (Fig. S1A to S1B, Fig. S2A to S2B, and Fig. S3A to S3B). In contrast, mHSPC patients with lower LMR exhibited markedly prolonged CFS (mCFS: 19.4 mo vs. 13.0 mo, *P* = 0.005) and OS (mOS: 81.7 mo vs. 58.0 mo, *P* = 0.007) (Fig. S4A and Fig. S4B). Furthermore, multivariate analysis confirmed that NLR and dNLR were independent prognosticators of CFS and OS in mHSPC cohort (Table S3 and Table S4).

In mCRPC cohort, patients with elevated NLR, dNLR and PLR showed a reduced PSA response rate (PSA response rate for NLR: 49.4% [43/87] vs. 74.6% [53/71], *P* = 0.001; PSA response rate for dNLR: 51.3% [39/76] vs. 69.5% [57/82], *P* = 0.019; PSA response rate for PLR: 53.1% [51/96] vs. 72.6% [45/62], *P* = 0.014;), significantly faster PSA progression (mPSA-PFS for NLR: 7.2 mo vs. 12.8 mo, *P* = 0.007; mPSA-PFS for dNLR: 5.1 mo vs. 11.2 mo, *P* < 0.001; mPSA-PFS for PLR: 8.6 mo vs. 14.0 mo, *P* = 0.009) and shorter OS (mOS for NLR: 32.3 mo vs. 62.5 mo, *P* < 0.001; mOS for dNLR: 25.9 mo vs. 41.3 mo, *P* = 0.002; mOS for PLR: 29.4 mo vs. 56.7 mo, *P* = 0.007) than those with lower indices, respectively (Fig. S1C to S1E, Fig. S2C to S2E, and Fig. S3C to S3E). Conversely, the PSA response rate (72.9% [70/96] vs. 41.9% [26/62], *P* < 0.001), PSA progression (mPSA-PFS: 12.3 mo vs. 5.7 mo, *P* < 0.001) and OS (mOS: 45.3 mo vs. 26.6 mo, *P* < 0.001) of mCRPC patients with lower LMR were significantly better than those with higher LMR (Fig. S4C to S4E). Multivariate analysis suggested NLR, dNLR, LMR, and PLR were independent prognosticators of PSA-PFS and OS (Table S5 and Table S6).

Similarly to SII and SIRI, we conducted supplementary Cox regression analyses, treating NLR, dNLR, LMR, and PLR as continuous variables in mHSPC and mCRPC patients to demonstrate their predictive efficacy (Tables S7, S8, S9, and S10).

**Fig. 5:**
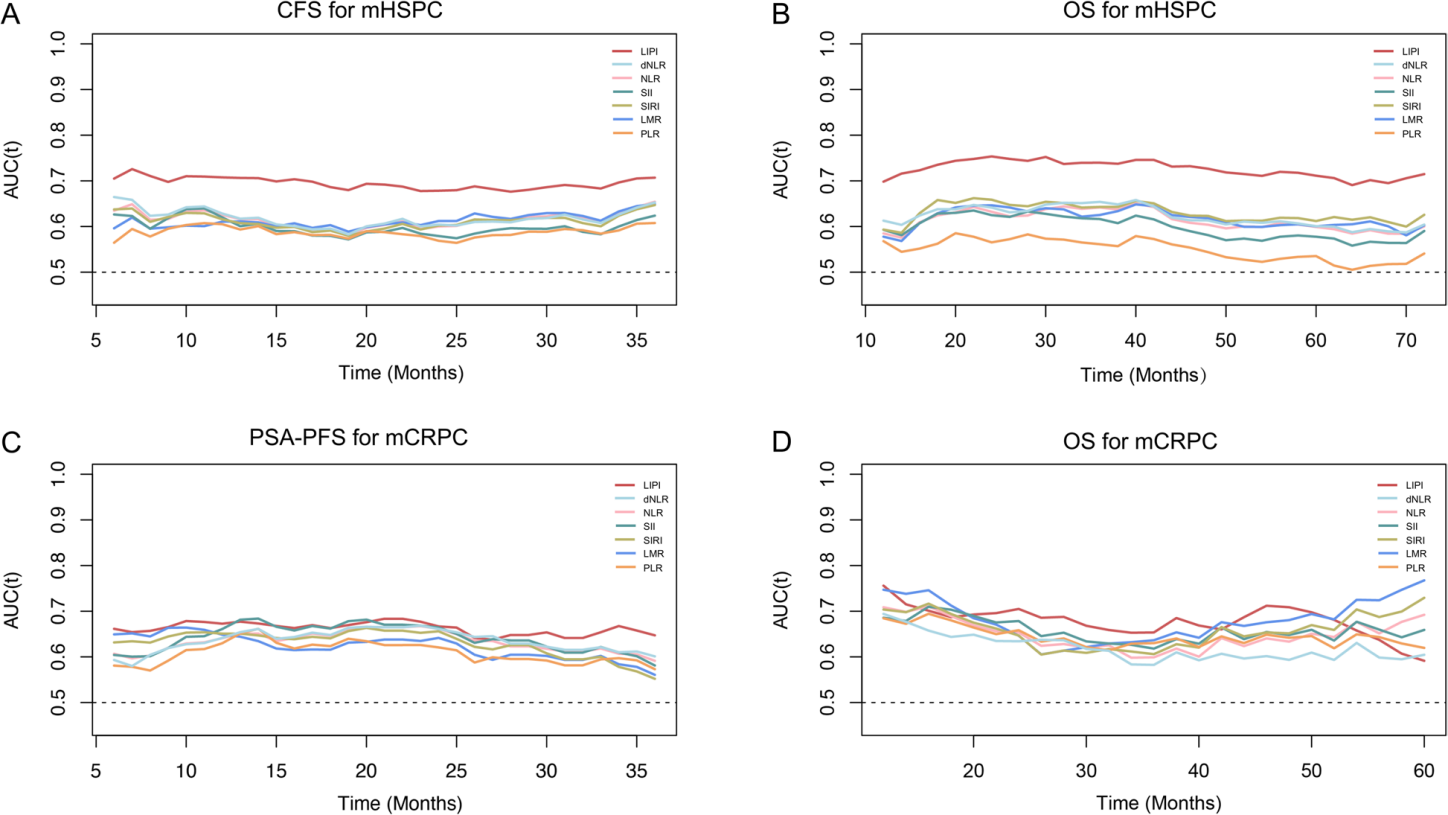
The time-dependent ROC to evaluate the predictive power of NLR, dNLR, LMR, PLR, SII, SIRI and LIPI in mHSPC and mCRPC cohorts. ROC analyses and AUC values of the seven indices: **A** CFS for mHSPC cohort, **B** OS for mHSPC cohort, **C** PSA-PFS for mCRPC cohort, and **D** OS for mCRPC cohort. NLR: neutrophil to lymphocyte ratio; dNLR: derived neutrophil to lymphocyte ratio; LMR: lymphocyte to monocyte ratio; PLR: platelet to lymphocyte ratio; SII: systemic immune inflammation index; SIRI: systemic inflammation response index; LIPI: lung immune prognostic index; mHSPC: metastatic hormone-sensitive prostate cancer; mCRPC: metastatic castration-resistant prostate cancer; ROC: receiver operating characteristic curve; AUC: area under the curve; CFS: castration-resistant prostate cancer-free survival; OS: overall survival; PSA-PFS: prostate-specific antigen progression-free survival

The predictive performance of each immune-inflammatory index was assessed using the AUC in time-dependent ROC curve analysis and C-index calculation. In mHSPC cohort, as illustrated in Fig. 5, LIPI consistently exhibited higher AUC values compared to NLR, dNLR, LMR, PLR, SII and SIRI for predicting CFS (Fig. 5A) and OS (Fig. 5B). Additionally, the C-indices of these indices for CFS, in descending order, were 0.643 (LIPI), 0.574 (NLR), 0.571 (dNLR), 0.568 (SIRI), 0.565 (SII), 0.559 (LMR) and 0.557 (PLR). For OS, the C-indices were 0.686 (LIPI), 0.602 (dNLR), 0.599 (SIRI), 0.592 (NLR), 0.590 (SII), 0.569 (LMR) and 0.548 (PLR), respectively (Table 3). These findings suggest that LIPI significantly outperformed the other six indices in predictive performance within the mHSPC cohort.

**Table 3:** C-index for seven immune-inflammatory indices in mHSPC and mCRPC cohorts

	C-index in mHSPC		C-index in mCRPC	
	**CFS**	**OS**	**PSA-PFS**	**OS**
**NLR**	0.574	0.592	0.577	0.595
**dNLR**	0.571	0.602	0.567	0.562
**LMR**	0.559	0.569	0.584	0.602
**PLR**	0.557	0.548	0.556	0.580
**SII**	0.565	0.590	0.586	0.620
**SIRI**	0.568	0.599	0.585	0.588
**LIPI**	0.643	0.686	0.617	0.640

*mHSPC* metastatic hormone-sensitive prostate cancer, *mCRPC* metastatic castration-resistant prostate cancer, *CFS* Castration-resistant prostate cancer-free survival, *OS* Overall survival, *PSA* Prostate-specific antigen, *PSA-PFS* Prostate-specific antigen progression-free survival, *NLR* Neutrophil to lymphocyte ratio, *dNLR* Derived neutrophil to lymphocyte ratio, *LMR* Lymphocyte to monocyte ratio, *PLR* Platelet to lymphocyte ratio, *SII* Systemic immune inflammation index, *SIRI* Systemic inflammation response index, *LIPI* Lung immune prognostic index

In the mCRPC cohort, unlike in the mHSPC cohort, LIPI did not consistently exhibit higher AUC values compared to the other indices at all time points for predicting PSA-PFS (Fig. 5C) and OS (Fig. 5D). Additionally, the C-indices of these indices for PSA-PFS, in descending order, were 0.617 (LIPI), 0.586 (SII), 0.585 (SIRI), 0.584 (PLR), 0.577 (NLR), 0.567 (dNLR) and 0.556 (LMR). For OS, the C-indices were 0.640 (LIPI), 0.620 (SII), 0.602 (PLR), 0.595 (NLR), 0.588 (SIRI), 0.580 (LMR) and 0.562 (dNLR), respectively (Table 3). These findings indicate that the notable predictive advantage of LIPI over other indices in the mHSPC stage diminished in the mCRPC stage.

## Discussion

Recently, several novel immune-inflammatory indices, such as LIPI, SII and SIRI, have demonstrated prognostic value in various types of cancers. This study firstly validated the applicability of LIPI, SII and SIRI in mHSPC and mCRPC. Simultaneously, we conducted a comprehensive comparison of the predictive performance of seven immune-inflammatory indices—NLR, dNLR, PLR, LMR, SII, SIRI, and LIPI—in advanced prostate cancer. Our results showed that LIPI had a relatively stronger discriminative ability than NLR, dNLR, PLR, LMR, SII and SIRI in mHSPC, while the predictive power of these seven immune-inflammatory indices was comparable in mCRPC. In summary, our comprehensive analysis of the prognostic value of immune-inflammatory indices in mPCa enhances clinicians' ability to effectively use laboratory test results for patient prognosis in clinical practice. Additionally, it may serve as a foundational reference for developing clinical prognostic models for mPCa and for screening patient baselines in clinical trials.

On the pan-cancer level, inflammation and immunity have a complex and inseparable relationship with tumors [21, 22]. Inflammation is an immune response to tissue damage or infection, but it can also promote cancer initiation and progression in various ways, such as activating carcinogens, inducing gene mutations, enhancing angiogenesis, inhibiting apoptosis, stimulating tumor cell proliferation and migration [23, 24]. Moreover, inflammation could modulate the tumor microenvironment to favor tumor cell survival and escape from immune system clearance [25–27]. Furthermore, inflammation could stimulate tumor cells to express immune checkpoint molecules, such as programmed cell death 1 ligand 1 (PD-L1), thereby inhibiting T lymphocyte activity and proliferation [26, 28]. Regarding PCa, inflammation drives prostate cancer by inducing DNA damage, activating oncogenic pathways, and generating "inflammatory storms" that enhance mutation and immune evasion, leading to prostate cancer progression [4]. In summary, the relationship between inflammation and immunity and tumors is complex and close [29, 30]. The various immune-inflammatory parameters in this study reflect the inflammatory and immune status of tumor patients in different forms and emphases. Meanwhile, the relationship between inflammation and immunity and tumors is not limited to a single type of tumor, which also explains the versatility of many immune-inflammatory parameters in various tumors.

Notably, Among the seven indices in this study, LIPI demonstrated superior predictive power in mHSPC cohort. The parameter composition might explain why LIPI has an advantage in prognostic prediction. The key difference between LIPI and the other six indices is that it incorporates LDH as a parameter. LDH is not only an enzyme related to inflammation, but also an enzyme related to metabolism. Compared with normal tissues, rapidly proliferating tumors may undergo more anaerobic glycolysis, and thus LDH as a glycolytic enzyme may be more expressed. In fact, high levels of LDH have been proven to be associated with malignant progression of various tumors, including prostate cancer [31, 32]. Another point of interest is that the notable predictive advantage of LIPI over other indices in the mHSPC stage diminishes in the mCRPC stage. This could be because patients at the mCRPC stage have typically undergone prolonged anti-tumor treatments, which can adversely affect liver function due to previous pharmacological interventions, thereby causing fluctuations in LDH levels and consequently diminishing the predictive efficacy of LIPI.

The advantage of the immune-inflammatory indices involved in this study is that they are all simple and non-invasive clinical parameters that can be easily calculated by only using the patient’s blood routine and biochemical routine tests. In clinical practice, these convenient and practical parameters can enable clinicians to quickly assess the prognosis of patients. However, given that these indices are derived from hematological tests, their predictive efficacy in cancer patients can be influenced by infectious diseases, hematological disorders, and certain metabolic diseases. This underscores the need for caution when using immune-inflammatory indices to predict cancer prognosis in clinical practice or subsequent research. Special attention should be paid to patients' underlying or concurrent diseases to avoid misinterpretation.

There are some limitations in this study. First, this is a single-center retrospective study with a relatively small sample size. The findings and results of this study need to be verified by studies with larger sample sizes. Second, this study did not assess the impact of dynamic changes of immune-inflammatory parameters due to a lack of data. Third, although we took care to exclude patients with diagnosed infectious diseases from our study based on their medical records, there might still be a small number of cancer patients with potential infectious diseases included in this retrospective study, which represents a limitation. Finally, there is no consensus on the optimal cutoff values for each parameter. Some studies choose the median value while others choose based on the principle of maximizing the Youden index like us. The optimal cutoff values still need to be determined by follow-up studies.

## Conclusion

In conclusion, this study first confirmed the prognostic value of SII, SIRI and LIPI in mHSPC and mCRPC, and compared the predictive performance of several immune-inflammatory parameters, including NLR, dNLR, PLR, SII, SIRI and LIPI. Among these indices, LIPI exhibited a significant discriminative ability in the mHSPC stage compared to the other indices, but this notable advantage diminishes in the mCRPC stage. These simple and non-invasive indices facilitate clinicians in quickly assessing the prognosis of patients.

### Supplementary Information

Supplementary Material 1.

Supplementary Material 2.

Supplementary Material 3.

Supplementary Material 4.

Supplementary Material 5.

Supplementary Material 6.

Supplementary Material 7.

Supplementary Material 8.

Supplementary Material 9.

Supplementary Material 10.

Supplementary Material 11: Fig. S1. The prognostic value of NLR in mHSPC and mCRPC cohorts. (A) Kaplan–Meier curves of CFS for mHSPC cohort; (B) Kaplan–Meier curves of OS for mHSPC cohort; (C) Kaplan–Meier curves of PSA-PFS for mCRPC cohort; (D) Kaplan–Meier curves of OS for mCRPC cohort; (E) PSA response rate for mCRPC cohort. NLR: neutrophil to lymphocyte ratio; mHSPC: metastatic hormone-sensitive prostate cancer; mCRPC: metastatic castration-resistant prostate cancer; CFS: castration-resistant prostate cancer-free survival; OS: overall survival; PSA: prostate-specific antigen; PSA-PFS: prostate-specific antigen progression-free survival.

Supplementary Material 12: Fig. S2. The prognostic value of dNLR in mHSPC and mCRPC cohorts. (A) Kaplan–Meier curves of CFS for mHSPC cohort; (B) Kaplan–Meier curves of OS for mHSPC cohort; (C) Kaplan–Meier curves of PSA-PFS for mCRPC cohort; (D) Kaplan–Meier curves of OS for mCRPC cohort; (E) PSA response rate for mCRPC cohort. dNLR: derived neutrophil to lymphocyte ratio; mHSPC: metastatic hormone-sensitive prostate cancer; mCRPC: metastatic castration-resistant prostate cancer; CFS: castration-resistant prostate cancer-free survival; OS: overall survival; PSA: prostate-specific antigen; PSA-PFS: prostate-specific antigen progression-free survival.

Supplementary Material 13: Fig. S3. The prognostic value of PLR in mHSPC and mCRPC cohorts. (A) Kaplan–Meier curves of CFS for mHSPC cohort; (B) Kaplan–Meier curves of OS for mHSPC cohort; (C) Kaplan–Meier curves of PSA-PFS for mCRPC cohort; (D) Kaplan–Meier curves of OS for mCRPC cohort; (E) PSA response rate for mCRPC cohort. PLR: platelet to lymphocyte ratio; mHSPC: metastatic hormone-sensitive prostate cancer; mCRPC: metastatic castration-resistant prostate cancer; CFS: castration-resistant prostate cancer-free survival; OS: overall survival; PSA: prostate-specific antigen; PSA-PFS: prostate-specific antigen progression-free survival.

Supplementary Material 14: Fig. S4. The prognostic value of LMR in mHSPC and mCRPC cohorts. (A) Kaplan–Meier curves of CFS for mHSPC cohort; (B) Kaplan–Meier curves of OS for mHSPC cohort; (C) Kaplan–Meier curves of PSA-PFS for mCRPC cohort; (D) Kaplan–Meier curves of OS for mCRPC cohort; (E) PSA response rate for mCRPC cohort. LMR: lymphocyte to monocyte ratio; mHSPC: metastatic hormone-sensitive prostate cancer; mCRPC: metastatic castration-resistant prostate cancer; CFS: castration-resistant prostate cancer-free survival; OS: overall survival; PSA: prostate-specific antigen; PSA-PFS: prostate-specific antigen progression-free survival.

## Data Availability

The dataset analyzed for results of this study are available from the corresponding author upon reasonable request.

## Abbreviations

PCa: Prostate cancer
CRPC: Castration-resistant prostate cancer
mPCa: Metastatic prostate cancer
mHSPC: Metastatic hormone-sensitive prostate cancer
mCRPC: Metastatic castration-resistant prostate cancer
NLR: Neutrophil to lymphocyte ratio
dNLR: Derived neutrophil to lymphocyte ratio
LMR: Lymphocyte to monocyte ratio
PLR: Platelet to lymphocyte ratio
SII: Systemic immune inflammation index
SIRI: Systemic inflammation response index
LIPI: Lung immune prognostic index
LDH: Lactate dehydrogenase
WBC: White blood cell
PLT: Platelet
ANC: Absolute neutrophil count
ALC: Absolute lymphocyte count
AMC: Absolute monocyte count
PSA: Prostate-specific antigen
GS: Gleason score
ECOG: Eastern Cooperative Oncology Group
HGB: Hemoglobin
ALP: Alkaline phosphatase
CFS: Castration-resistant prostate cancer-free survival
OS: Overall survival
PSA-PFS: Prostate-specific antigen progression-free survival
AUC: Area under the curve
ROC: Receiver operating characteristic curve
PD-L1: Programmed cell death 1 ligand 1
HR: Hazard ratio
CI: Confidence interval

## References

[1] Siegel RL, Miller KD, Wagle NS, Jemal A (2023). Cancer statistics, 2023. CA Cancer J Clin.

[2] Zhu Y, Mo M, Wei Y, Wu J, Pan J, Freedland SJ (2021). Epidemiology and genomics of prostate cancer in Asian men. Nat Rev Urol.

[3] Health Commission Of The People's Republic Of China N (2022). National guidelines for diagnosis and treatment of prostate cancer 2022 in China (English version). Chin J Cancer Res.

[4] de Bono JS, Guo C, Gurel B, De Marzo AM, Sfanos KS, Mani RS (2020). Prostate carcinogenesis: inflammatory storms. Nat Rev Cancer.

[5] Huang L, LaBonte MJ, Craig SG, Finn SP, Allott EH. Inflammation and Prostate Cancer: A Multidisciplinary Approach to Identifying Opportunities for Treatment and Prevention. Cancers (Basel). 2022;14(6)1367. 10.3390/cancers14061367 PMC8946208 35326519

[6] Lolli C, Caffo O, Scarpi E, Aieta M, Conteduca V, Maines F (2016). Systemic immune-inflammation index predicts the clinical outcome in patients with mCRPC treated with abiraterone. Front Pharmacol.

[7] Bauckneht M, Rebuzzi SE, Signori A, Frantellizzi V, Murianni V, Lodi Rizzini E (2022). The prognostic power of inflammatory indices and clinical factors in metastatic castration-resistant prostate cancer patients treated with radium-223 (BIO-Ra study). Eur J Nucl Med Mol Imaging.

[8] Guan Y, Xiong H, Feng Y, Liao G, Tong T, Pang J (2020). Revealing the prognostic landscape of neutrophil-to-lymphocyte ratio and platelet-to-lymphocyte ratio in metastatic castration-resistant prostate cancer patients treated with abiraterone or enzalutamide: a meta-analysis. Prostate Cancer Prostatic Dis.

[9] Mezquita L, Auclin E, Ferrara R, Charrier M, Remon J, Planchard D (2018). Association of the lung immune prognostic index with immune checkpoint inhibitor outcomes in patients with advanced non-small cell lung cancer. JAMA Oncol.

[10] Kazandjian D, Gong Y, Keegan P, Pazdur R, Blumenthal GM (2019). Prognostic value of the lung immune prognostic index for patients treated for metastatic non-small cell lung cancer. JAMA Oncol.

[11] Feng JF, Zhao JM, Chen S, Chen QX (2021). Prognostic significance of the lung immune prognostic index in patients with resected esophageal squamous cell carcinoma. Cancer Manag Res.

[12] Obayashi K, Miki J, Fukuokaya W, Yanagisawa T, Kimura S, Tsuzuki S (2022). The prognostic value of the preoperative lung immune prognostic index in patients with urothelial bladder cancer undergoing radical cystectomy. Int J Clin Oncol.

[13] Hu B, Yang XR, Xu Y, Sun YF, Sun C, Guo W (2014). Systemic immune-inflammation index predicts prognosis of patients after curative resection for hepatocellular carcinoma. Clin Cancer Res.

[14] Geng Y, Zhu D, Wu C, Wu J, Wang Q, Li R (2018). A novel systemic inflammation response index (SIRI) for predicting postoperative survival of patients with esophageal squamous cell carcinoma. Int Immunopharmacol.

[15] Xie QK, Chen P, Hu WM, Sun P, He WZ, Jiang C (2018). The systemic immune-inflammation index is an independent predictor of survival for metastatic colorectal cancer and its association with the lymphocytic response to the tumor. J Transl Med.

[16] Qi Q, Zhuang L, Shen Y, Geng Y, Yu S, Chen H (2016). A novel systemic inflammation response index (SIRI) for predicting the survival of patients with pancreatic cancer after chemotherapy. Cancer.

[17] Dong J, Sun Q, Pan Y, Lu N, Han X, Zhou Q (2021). Pretreatment systemic inflammation response index is predictive of pathological complete response in patients with breast cancer receiving neoadjuvant chemotherapy. BMC Cancer.

[18] Huang C, Wang M, Chen L, Wang H, Huang D, Shi J (2023). The pretherapeutic systemic inflammation score is a prognostic predictor for elderly patients with oesophageal cancer: a case control study. BMC Cancer.

[19] Chao B, Ju X, Zhang L, Xu X, Zhao Y (2020). A novel prognostic marker systemic inflammation response index (siri) for operable cervical cancer patients. Front Oncol.

[20] Cornford P, van den Bergh RCN, Briers E, Van den Broeck T, Cumberbatch MG, De Santis M (2021). EAU-EANM-ESTRO-ESUR-SIOG guidelines on prostate cancer. part ii-2020 update: treatment of relapsing and metastatic prostate cancer. Eur Urol..

[21] Gong YT, Zhang LJ, Liu YC, Tang M, Lin JY, Chen XY (2023). Neutrophils as potential therapeutic targets for breast cancer. Pharmacol Res.

[22] Greten FR, Grivennikov SI (2019). Inflammation and cancer: triggers, mechanisms, and consequences. Immunity.

[23] Coussens LM, Werb Z (2002). Inflammation and cancer. Nature.

[24] Yan L, Li W, Chen F, Wang J, Chen J, Chen Y (2023). Inflammation as a mediator of microbiome dysbiosis-associated DNA methylation changes in gastric premalignant lesions. Phenomics.

[25] Kuo C-L, Ponneri Babuharisankar A, Lin Y-C, Lien H-W, Lo YK, Chou H-Y (2022). Mitochondrial oxidative stress in the tumor microenvironment and cancer immunoescape: foe or friend?. J Biomed Sci.

[26] Khalaf K, Hana D, Chou JT, Singh C, Mackiewicz A, Kaczmarek M (2021). Aspects of the tumor microenvironment involved in immune resistance and drug resistance. Front Immunol.

[27] Jiang M, Gu D, Liu F, Lin C, Tian L (2023). Transforming cancer cells for long-term living with cancer: an inspiring new approach. Chin J Cancer Res.

[28] Lamplugh Z, Fan Y (2021). Vascular microenvironment, tumor immunity and immunotherapy. Front Immunol.

[29] Feng D, Xiong Q, Wei Q, Yang L (2023). Cellular landscape of tumour microenvironment in prostate cancer. Immunology.

[30] Elinav E, Nowarski R, Thaiss CA, Hu B, Jin C, Flavell RA (2013). Inflammation-induced cancer: crosstalk between tumours, immune cells and microorganisms. Nat Rev Cancer.

[31] Petrelli F, Cabiddu M, Coinu A, Borgonovo K, Ghilardi M, Lonati V (2015). Prognostic role of lactate dehydrogenase in solid tumors: a systematic review and meta-analysis of 76 studies. Acta Oncol.

[32] Li F, Xiang H, Pang Z, Chen Z, Dai J, Chen S (2020). Association between lactate dehydrogenase levels and oncologic outcomes in metastatic prostate cancer: a meta-analysis. Cancer Med.

