# Supplementary material for: Analysis of the immune-inflammatory indices for patients with metastatic hormone-sensitive and castration-resistant prostate cancer
Source: BMC Cancer. 2024 Jul 9;24:817. doi: 10.1186/s12885-024-12593-z (PMC11232225; doi:10.1186/s12885-024-12593-z)
Supplement: Supplementary file 1 — Supplementary Material 1. [file 12885_2024_12593_MOESM1_ESM.docx]

**Table S1. Sequential treatment for mCRPC patients.**

| **First-line therapy** | **Abiraterone** |
| --- | --- |
| Number of patients received sequential treatment | 51 (32.3%) |
| Docetaxel | 35 (22.2%) |
| Oxaliplatin | 2 (1.3%) |
| Proxalutamide | 5 (3.2%) |
| Enzalutamide | 8 (5.1%) |
| Rezvilutamide | 3 (1.9%) |
| Apalutamide | 3 (1.9%) |
| Olaparib | 13 (8.2%) |
| Pembrolizumab | 3 (1.9%) |
| ≥3 Therapies | 17 (10.8%) |

mCRPC = metastatic castration-resistant prostate cancer
