# Supplementary material for: Analysis of the immune-inflammatory indices for patients with metastatic hormone-sensitive and castration-resistant prostate cancer
Source: BMC Cancer. 2024 Jul 9;24:817. doi: 10.1186/s12885-024-12593-z (PMC11232225; doi:10.1186/s12885-024-12593-z)
Supplement: Supplementary file 2 — Supplementary Material 2. [file 12885_2024_12593_MOESM2_ESM.docx]

| Indices | mHSPC | | mCRPC | | |
| --- | --- | --- | --- | --- | --- |
|  | **CFS** | **OS** | **PSA response rate** | **PSA-PFS** | **OS** |
| NLR | 2.55 | 3.33 | 2.27 | 2.87 | 2.04 |
| dNLR | 2.03 | 2.96 | 1.73 | 2.74 | 2.00 |
| LMR | 3.74 | 4.16 | 3.09 | 2.57 | 2.41 |
| PLR | 163.22 | 123.81 | 97.57 | 112.86 | 112.86 |
| SII | 449.49 | 550.96 | 343.87 | 349.00 | 374.00 |
| SIRI | 1.28 | 1.83 | 1.21 | 2.12 | 0.87 |

**Table S2. The optimal cut-off points.**

mHSPC = metastatic hormone-sensitive prostate cancer; mCRPC = metastatic castration-resistant prostate cancer; NLR = neutrophil to lymphocyte ratio; dNLR = derived neutrophil to lymphocyte ratio; LMR = lymphocyte to monocyte ratio; PLR = platelet to lymphocyte ratio; SII = systemic immune inflammation index; SIRI = systemic inflammation response index; CFS = castration-resistant prostate cancer-free survival; OS = overall survival; PSA = prostate-specific antigen; PSA-PFS = prostate-specific antigen progression-free survival.
