# Supplementary material for: Analysis of the immune-inflammatory indices for patients with metastatic hormone-sensitive and castration-resistant prostate cancer
Source: BMC Cancer. 2024 Jul 9;24:817. doi: 10.1186/s12885-024-12593-z (PMC11232225; doi:10.1186/s12885-024-12593-z)
Supplement: Supplementary file 3 — Supplementary Material 3. [file 12885_2024_12593_MOESM3_ESM.docx]

**Table S3. Univariate and multivariate analyses of CFS in mHSPC cohort.**

|  | **Univariate analysis** | | **Multivariate analysis** | |
| --- | --- | --- | --- | --- |
|  | **HR (95% CI)** | **P** | **HR (95% CI)** | **P** |
| **Age (y), ≥72 vs. <72** | 0.84 (0.67-1.04) | 0.111 | - | - |
| **ECOG, ≥2 vs. <0-1** | 1.29 (0.94-1.77) | 0.110 | - | - |
| **ISUP group, 5 vs. 1-3** | 2.06 (1.49-2.85) | <0.001 | 1.96 (1.41-2.73) | <0.001 |
| **ISUP group, 5 vs. 4** | 1.71 (1.26-2.31) | 0.001 | 1.63 (1.20-2.21) | 0.002 |
| **VM, yes vs. no** | 1.28 (0.92-1.78) | 0.146 | - | - |
| **PSA (ng/ml), ≥100 vs. <100** | 1.27 (1.01-1.59) | 0.040 | 1.06 (0.84-1.33) | 0.645 |
| **HGB (g/L), <120 vs. ≥120** | 2.43 (1.92-3.09) | <0.001 | 1.81 (1.39-2.35) | <0.001 |
| **ALP (IU/L), ≥160 vs. <160** | 2.32 (1.83-2.93) | <0.001 | 1.59 (1.22-2.08) | 0.001 |
| **LDH (IU/L), ≥220 vs. <220** | 2.50 (2.00-3.13) | <0.001 | 1.78 (1.39-2.28) | <0.001 |
| **NLR, ≥2.55 vs. <2.55** | 1.63 (1.30-2.03) | <0.001 | 1.44 (1.15-1.81) | 0.002^#^ |
| **dNLR, ≥2.03 vs. <2.03** | 1.60 (1.28-1.99) | <0.001 | 1.43 (1.15-1.80) | 0.002^#^ |
| **LMR, ≥3.74 vs. <3.74** | 0.73 (0.59-0.91) | 0.005 | 0.84 (0.67-1.05) | 0.133^#^ |
| **PLR, ≥163.22 vs. <163.22** | 1.58 (1.24-2.01) | <0.001 | 1.24 (0.97-1.59) | 0.093^#^ |
| **SII, ≥449.49 vs. <449.49** | 1.54 (1.24-1.93) | <0.001 | 1.35 (1.08-1.70) | 0.009^#^ |
| **SIRI, ≥1.28 vs. <1.28** | 1.59 (1.27-1.98) | <0.001 | 1.45 (1.16-1.81) | 0.001^#^ |
| **LIPI-Poor vs. Good** | 5.29 (3.82-7.30) | <0.001 | 5.81 (3.70-9.09) | <0.001* |
| **LIPI-Poor vs. Inter.** | 2.72 (1.97-3.75) | <0.001 | 2.64 (1.75-3.97) | <0.001* |

y = year; mHSPC = metastatic hormone-sensitive prostate cancer; CFS = castration-resistant prostate cancer-free survival; HR = hazard ratio; CI = confidence interval; ECOG = Eastern Cooperative Oncology Group; ISUP = International Society of Urological Pathology; VM = Visceral metastasis; PSA = prostate-specific antigen; HGB = hemoglobin; ALP = alkaline phosphatase; LDH = lactate dehydrogenase; NLR = neutrophil to lymphocyte ratio; dNLR = derived neutrophil to lymphocyte ratio; LMR = lymphocyte to monocyte ratio; PLR = platelet to lymphocyte ratio; SII = systemic immune inflammation index; SIRI = systemic inflammation response index; LIPI: lung immune prognostic index. ^#^Adjusted for ISUP, PSA, HGB, ALP and LDH. *: Adjusted for ISUP, PSA, HGB and ALP.
