# Supplementary material for: Analysis of the immune-inflammatory indices for patients with metastatic hormone-sensitive and castration-resistant prostate cancer
Source: BMC Cancer. 2024 Jul 9;24:817. doi: 10.1186/s12885-024-12593-z (PMC11232225; doi:10.1186/s12885-024-12593-z)
Supplement: Supplementary file 4 — Supplementary Material 4. [file 12885_2024_12593_MOESM4_ESM.docx]

**Table S4. Univariate and multivariate analyses of OS in mHSPC cohort.**

|  | **Univariate analysis** | | **Multivariate analysis** | |
| --- | --- | --- | --- | --- |
|  | **HR (95% CI)** | **P** | **HR (95% CI)** | **P** |
| **Age (y), ≥72 vs. <72** | 1.03 (0.77-1.37) | 0.856 | - | - |
| **ECOG, ≥2 vs. <0-1** | 1.95 (1.36-2.80) | <0.001 | 1.73 (1.19-2.51) | 0.004 |
| **ISUP group, 5 vs. 1-3** | 2.00 (1.30-3.08) | 0.002 | 2.31 (1.48-3.62) | <0.001 |
| **ISUP group, 5 vs. 4** | 2.00 (1.30-3.09) | 0.002 | 1.74 (1.12-2.70) | 0.014 |
| **VM, yes vs. no** | 1.36 (0.90-2.06) | 0.145 | - | - |
| **PSA (ng/ml), ≥100 vs. <100** | 1.02 (0.76-1.37) | 0.902 | - | - |
| **HGB (g/L), <120 vs. ≥120** | 2.55 (1.88-3.47) | <0.001 | 2.05 (1.46-2.88) | <0.001 |
| **ALP (IU/L), ≥160 vs. <160** | 1.84 (1.35-2.49) | <0.001 | 1.01 (0.71-1.43) | 0.963 |
| **LDH (IU/L), ≥220 vs. <220** | 3.28 (2.44-4.41) | <0.001 | 2.53 (1.83-3.49) | <0.001 |
| **NLR, ≥3.33 vs. <3.33** | 1.93 (1.44-2.59) | <0.001 | 1.69 (1.25-2.28) | 0.001^#^ |
| **dNLR, ≥2.96 vs. <2.96** | 2.56 (1.89-3.48) | <0.001 | 2.24 (1.63-3.07) | <0.001^#^ |
| **LMR, ≥4.16 vs. <4.16** | 0.65 (0.48-0.89) | 0.007 | 0.76 (0.55-1.04) | 0.087^#^ |
| **PLR, ≥123.81 vs. <123.81** | 1.43 (1.07-1.91) | 0.016 | 1.06 (0.78-1.44) | 0.701^#^ |
| **SII, ≥550.96 vs. <550.96** | 1.90 (1.42-2.55) | <0.001 | 1.60 (1.19-2.15) | 0.002^#^ |
| **SIRI, ≥1.83 vs. <1.83** | 2.16 (1.61-2.91) | <0.001 | 1.82 (1.34-2.47) | <0.001^#^ |
| **LIPI-Poor vs. Good** | 7.52 (4.90-11.49) | <0.001 | 5.92 (3.77-9.26) | <0.001* |
| **LIPI-Poor vs. Inter.** | 2.72 (1.82-4.05) | <0.001 | 2.67 (1.78-4.02) | <0.001* |

y = year; mHSPC = metastatic hormone-sensitive prostate cancer; OS = overall survival; HR = hazard ratio; CI = confidence interval; ECOG = Eastern Cooperative Oncology Group; ISUP = International Society of Urological Pathology; VM = Visceral metastasis; PSA = prostate-specific antigen; HGB = hemoglobin; ALP = alkaline phosphatase; LDH = lactate dehydrogenase; NLR = neutrophil to lymphocyte ratio; dNLR = derived neutrophil to lymphocyte ratio; LMR = lymphocyte to monocyte ratio; PLR = platelet to lymphocyte ratio; SII = systemic immune inflammation index; SIRI = systemic inflammation response index; LIPI: lung immune prognostic index. ^#^Adjusted for ECOG, ISUP, HGB, ALP and LDH. *: Adjusted for ECOG, ISUP, HGB and ALP.
