# Supplementary material for: Analysis of the immune-inflammatory indices for patients with metastatic hormone-sensitive and castration-resistant prostate cancer
Source: BMC Cancer. 2024 Jul 9;24:817. doi: 10.1186/s12885-024-12593-z (PMC11232225; doi:10.1186/s12885-024-12593-z)
Supplement: Supplementary file 5 — Supplementary Material 5. [file 12885_2024_12593_MOESM5_ESM.docx]

**Table S5. Univariate and multivariate analyses of PSA-PFS in mCRPC cohort.**

|  | **Univariate analysis** | | **Multivariate analysis** | |
| --- | --- | --- | --- | --- |
|  | **HR (95% CI)** | **P** | **HR (95% CI)** | **P** |
| **Age (y), ≥72 vs. <72** | 0.92 (0.66-1.28) | 0.619 | - | - |
| **ECOG, ≥2 vs. <0-1** | 2.15 (1.15-4.00) | 0.016 | 2.18 (1.13-4.22) | 0.021 |
| **ISUP group, 5 vs. 1-3** | 1.63 (0.93-2.86) | 0.089 | - | - |
| **ISUP group, 5 vs. 4** | 1.26 (0.83-1.92) | 0.286 | - | - |
| **VM, yes vs. no** | 1.14 (0.69-1.87) | 0.618 | - | - |
| **PSA (ng/ml), ≥12 vs. <12** | 1.30 (0.93-1.82) | 0.125 | - | - |
| **HGB (g/L), <120 vs. ≥120** | 1.42 (1.01-2.01) | 0.046 | 1.43 (1.00-2.04) | 0.051 |
| **ALP (IU/L), ≥160 vs. <160** | 1.56 (1.07-2.26) | 0.021 | 1.34 (0.90-2.01) | 0.152 |
| **LDH (IU/L), ≥220 vs. <220** | 1.60 (1.14-2.24) | 0.006 | 1.50 (1.06-2.14) | 0.023 |
| **NLR, ≥2.87 vs. <2.87** | 1.58 (1.13-2.22) | 0.008 | 1.85 (1.30-2.64) | 0.001^#^ |
| **dNLR, ≥2.74 vs. <2.74** | 2.08 (1.39-3.11) | <0.001 | 2.10 (1.40-3.16) | <0.001^#^ |
| **LMR, ≥2.57 vs. <2.57** | 0.51 (0.36-0.74) | 0.001 | 0.44 (0.30-0.64) | <0.001^#^ |
| **PLR, ≥112.86 vs. <112.86** | 1.56 (1.12-2.19) | 0.009 | 1.55 (1.09-2.21) | 0.016^#^ |
| **SII, ≥349.00 vs. <349.00** | 1.72 (1.21-2.45) | 0.003 | 1.73 (1.20-2.50) | 0.003^#^ |
| **SIRI, ≥2.12 vs. <2.12** | 1.59 (1.14-2.23) | 0.007 | 1.59 (1.13-2.24) | 0.008^#^ |
| **LIPI-Poor vs. Good** | 3.79 (2.23-6.45) | <0.001 | 3.85 (2.25-6.58) | <0.001* |
| **LIPI-Poor vs. Inter.** | 2.41 (1.43-4.05) | 0.001 | 2.72 (1.60-4.63) | <0.001* |

y = year; mCRPC = metastatic castration-resistant prostate cancer; PSA-PFS = prostate-specific antigen progression-free survival; HR = hazard ratio; CI = confidence interval; ECOG = Eastern Cooperative Oncology Group; ISUP = International Society of Urological Pathology; VM = Visceral metastasis; PSA = prostate-specific antigen; HGB = hemoglobin; ALP = alkaline phosphatase; LDH = lactate dehydrogenase; NLR = neutrophil to lymphocyte ratio; dNLR = derived neutrophil to lymphocyte ratio; LMR = lymphocyte to monocyte ratio; PLR = platelet to lymphocyte ratio; SII = systemic immune inflammation index; SIRI = systemic inflammation response index; LIPI: lung immune prognostic index. ^#^Adjusted for ECOG, HGB, ALP and LDH. *: Adjusted for ECOG, HGB and ALP.
