# Supplementary material for: Analysis of the immune-inflammatory indices for patients with metastatic hormone-sensitive and castration-resistant prostate cancer
Source: BMC Cancer. 2024 Jul 9;24:817. doi: 10.1186/s12885-024-12593-z (PMC11232225; doi:10.1186/s12885-024-12593-z)
Supplement: Supplementary file 6 — Supplementary Material 6. [file 12885_2024_12593_MOESM6_ESM.docx]

**Table S6. Univariate and multivariate analyses of OS in mCRPC cohort.**

|  | **Univariate analysis** | | **Multivariate analysis** | |
| --- | --- | --- | --- | --- |
|  | **HR (95% CI)** | **P** | **HR (95% CI)** | **P** |
| **Age (y), ≥72 vs. <72** | 1.08 (0.72-1.62) | 0.704 | - | - |
| **ECOG, ≥2 vs. <0-1** | 2.27 (1.17-4.38) | 0.015 | 2.50 (1.21-5.17) | 0.013 |
| **ISUP group, 5 vs. 1-3** | 2.86 (1.24-6.62) | 0.014 | 3.46 (1.47-8.13) | 0.004 |
| **ISUP group, 5 vs. 4** | 1.19 (0.72-1.95) | 0.494 | 1.31 (0.78-2.19) | 0.307 |
| **VM, yes vs. no** | 1.74 (0.99-3.03) | 0.053 | - | - |
| **PSA (ng/ml), ≥12 vs. <12** | 1.93 (1.27-2.92) | 0.002 | 1.72 (1.09-2.71) | 0.020 |
| **HGB (g/L), <120 vs. ≥120** | 2.10 (1.39-3.17) | <0.001 | 1.75 (1.12-2.72) | 0.014 |
| **ALP (IU/L), ≥160 vs. <160** | 2.07 (1.31-3.27) | 0.002 | 0.96 (0.55-1.69) | 0.896 |
| **LDH (IU/L), ≥220 vs. <220** | 1.88 (1.24-2.83) | 0.003 | 1.80 (1.14-2.86) | 0.012 |
| **NLR, ≥2.04 vs. <2.04** | 2.19 (1.38-3.47) | 0.001 | 2.19 (1.35-3.56) | 0.002^#^ |
| **dNLR, ≥2.00 vs. <2.00** | 1.66 (1.10-2.50) | 0.016 | 2.01 (1.29-3.12) | 0.002^#^ |
| **LMR, ≥2.41 vs. <2.41** | 0.38 (0.24-0.58) | <0.001 | 0.33 (0.21-0.53) | <0.001^#^ |
| **PLR, ≥112.86 vs. <112.86** | 1.75 (1.16-2.64) | 0.007 | 1.87 (1.20-2.93) | 0.006^#^ |
| **SII, ≥374.00 vs. <374.00** | 2.25 (1.45-3.48) | <0.001 | 2.39 (1.52-3.76) | <0.001^#^ |
| **SIRI, ≥0.87 vs. <0.87** | 2.21 (1.40-3.48) | 0.001 | 2.55 (1.59-4.07) | <0.001^#^ |
| **LIPI-Poor vs. Good** | 4.98 (2.72-9.17) | <0.001 | 6.67 (3.44-12.8) | <0.001* |
| **LIPI-Poor vs. Inter.** | 3.16 (1.76-5.71) | <0.001 | 4.83 (2.56-9.09) | <0.001* |

y = year; mCRPC = metastatic castration-resistant prostate cancer; OS = overall survival; HR = hazard ratio; CI = confidence interval; ECOG = Eastern Cooperative Oncology Group; ISUP = International Society of Urological Pathology; VM = Visceral metastasis; PSA = prostate-specific antigen; HGB = hemoglobin; ALP = alkaline phosphatase; LDH = ; NLR = neutrophil to lymphocyte ratio; dNLR = derived neutrophil to lymphocyte ratio; LMR = lymphocyte to monocyte ratio; PLR = platelet to lymphocyte ratio; SII = systemic immune inflammation index; SIRI = systemic inflammation response index; LIPI: lung immune prognostic index. ^#^Adjusted for ECOG, ISUP, PSA, HGB, ALP and LDH. *: Adjusted for ECOG, ISUP, PSA, HGB, and ALP.
