# Supplementary material for: Analysis of the immune-inflammatory indices for patients with metastatic hormone-sensitive and castration-resistant prostate cancer
Source: BMC Cancer. 2024 Jul 9;24:817. doi: 10.1186/s12885-024-12593-z (PMC11232225; doi:10.1186/s12885-024-12593-z)
Supplement: Supplementary file 7 — Supplementary Material 7. [file 12885_2024_12593_MOESM7_ESM.docx]

**Table S7. Univariate and multivariate analyses of CFS in mHSPC cohort.**

|  | **Univariate analysis** | | **Multivariate analysis** | |
| --- | --- | --- | --- | --- |
|  | **HR (95% CI)** | **P** | **HR (95% CI)** | **P** |
| **Age (y), ≥72 vs. <72** | 0.84 (0.67-1.04) | 0.111 | - | - |
| **ECOG, ≥2 vs. <0-1** | 1.29 (0.94-1.77) | 0.110 | - | - |
| **ISUP group, 5 vs. 1-3** | 2.06 (1.49-2.85) | <0.001 | 1.92 (1.38-2.68) | <0.001 |
| **ISUP group, 5 vs. 4** | 1.71 (1.26-2.31) | 0.001 | 1.74 (1.28-2.35) | <0.001 |
| **VM, yes vs. no** | 1.28 (0.92-1.78) | 0.146 | - | - |
| **PSA (ng/ml), ≥100 vs. <100** | 1.27 (1.01-1.59) | 0.040 | 1.05 (0.83-1.32) | 0.703 |
| **HGB (g/L), <120 vs. ≥120** | 2.43 (1.92-3.09) | <0.001 | 1.83 (1.40-2.39) | <0.001 |
| **ALP (IU/L), ≥160 vs. <160** | 2.32 (1.83-2.93) | <0.001 | 1.65 (1.26-2.14) | <0.001 |
| **LDH (IU/L), ≥220 vs. <220** | 2.50 (2.00-3.13) | <0.001 | 1.77 (1.38-2.26) | <0.001 |
| **NLR (continuous variable)** | 1.04 (1.01-1.06) | 0.010 | 1.02 (0.99-1.05) | 0.232* |
| **dNLR (continuous variable)** | 1.07 (1.02-1.13) | 0.008 | 1.06 (1.00-1.12) | 0.064* |
| **LMR (continuous variable)** | 1.00 (1.00-1.00) | 0.626 | - | - |
| **PLR (continuous variable)** | 1.00 (1.00-1.00) | <0.001 | 1.00 (1.00-1.00) | 0.264* |
| **SII (continuous variable)** | 1.00 (1.00-1.00) | 0.002 | 1.00 (1.00-1.00) | 0.072* |
| **SIRI (continuous variable)** | 1.04 (1.01-1.08) | 0.009 | 1.03 (1.00-1.07) | 0.096* |

y = year; mHSPC = metastatic hormone-sensitive prostate cancer; CFS = castration-resistant prostate cancer-free survival; HR = hazard ratio; CI = confidence interval; ECOG = Eastern Cooperative Oncology Group; ISUP = International Society of Urological Pathology; VM = Visceral metastasis; PSA = prostate-specific antigen; HGB = hemoglobin; ALP = alkaline phosphatase; LDH = lactate dehydrogenase; NLR = neutrophil to lymphocyte ratio; dNLR = derived neutrophil to lymphocyte ratio; LMR = lymphocyte to monocyte ratio; PLR = platelet to lymphocyte ratio; SII = systemic immune inflammation index; SIRI = systemic inflammation response index. *Adjusted for ISUP, PSA, HGB, ALP and LDH.
