# Supplementary material for: Analysis of the immune-inflammatory indices for patients with metastatic hormone-sensitive and castration-resistant prostate cancer
Source: BMC Cancer. 2024 Jul 9;24:817. doi: 10.1186/s12885-024-12593-z (PMC11232225; doi:10.1186/s12885-024-12593-z)
Supplement: Supplementary file 8 — Supplementary Material 8. [file 12885_2024_12593_MOESM8_ESM.docx]

**Table S8. Univariate and multivariate analyses of OS in mHSPC cohort.**

|  | **Univariate analysis** | | **Multivariate analysis** | |
| --- | --- | --- | --- | --- |
|  | **HR (95% CI)** | **P** | **HR (95% CI)** | **P** |
| **Age (y), ≥72 vs. <72** | 1.03 (0.77-1.37) | 0.856 | - | - |
| **ECOG, ≥2 vs. <0-1** | 1.95 (1.36-2.80) | <0.001 | 1.67 (1.16-2.42) | 0.006 |
| **ISUP group, 5 vs. 1-3** | 2.00 (1.30-3.08) | 0.002 | 2.11 (1.36-3.27) | 0.001 |
| **ISUP group, 5 vs. 4** | 2.00 (1.30-3.09) | 0.002 | 1.89 (1.22-2.92) | 0.005 |
| **VM, yes vs. no** | 1.36 (0.90-2.06) | 0.145 | - | - |
| **PSA (ng/ml), ≥100 vs. <100** | 1.02 (0.76-1.37) | 0.902 | - | - |
| **HGB (g/L), <120 vs. ≥120** | 2.55 (1.88-3.47) | <0.001 | 2.08 (1.48-2.94) | <0.001 |
| **ALP (IU/L), ≥160 vs. <160** | 1.84 (1.35-2.49) | <0.001 | 1.03 (0.72-1.46) | 0.888 |
| **LDH (IU/L), ≥220 vs. <220** | 3.28 (2.44-4.41) | <0.001 | 2.60 (1.88-3.59) | <0.001 |
| **NLR (continuous variable)** | 1.04 (1.01-1.08) | 0.009 | 1.03 (0.99-1.07) | 0.139* |
| **dNLR (continuous variable)** | 1.10 (1.04-1.17) | 0.002 | 1.09 (1.01-1.16) | 0.022* |
| **LMR (continuous variable)** | 0.86 (0.79-0.95) | 0.001 | 0.90 (0.83-0.96) | 0.021* |
| **PLR (continuous variable)** | 1.00 (1.00-1.00) | 0.069 | 1.00 (1.00-1.00) | 0.759* |
| **SII (continuous variable)** | 1.00 (1.00-1.00) | 0.010 | 1.00 (1.00-1.00) | 0.077* |
| **SIRI (continuous variable)** | 1.09 (1.05-1.13) | <0.001 | 1.06 (1.02-1.11) | 0.003* |

y = year; mHSPC = metastatic hormone-sensitive prostate cancer; OS = overall survival; HR = hazard ratio; CI = confidence interval; ECOG = Eastern Cooperative Oncology Group; ISUP = International Society of Urological Pathology; VM = Visceral metastasis; PSA = prostate-specific antigen; HGB = hemoglobin; ALP = alkaline phosphatase; LDH = lactate dehydrogenase; NLR = neutrophil to lymphocyte ratio; dNLR = derived neutrophil to lymphocyte ratio; LMR = lymphocyte to monocyte ratio; PLR = platelet to lymphocyte ratio; SII = systemic immune inflammation index; SIRI = systemic inflammation response index. *Adjusted for ECOG, ISUP, HGB, ALP and LDH.
