# Supplementary material for: Analysis of the immune-inflammatory indices for patients with metastatic hormone-sensitive and castration-resistant prostate cancer
Source: BMC Cancer. 2024 Jul 9;24:817. doi: 10.1186/s12885-024-12593-z (PMC11232225; doi:10.1186/s12885-024-12593-z)
Supplement: Supplementary file 9 — Supplementary Material 9. [file 12885_2024_12593_MOESM9_ESM.docx]

**Table S9. Univariate and multivariate analyses of PSA-PFS in mCRPC cohort.**

|  | **Univariate analysis** | | **Multivariate analysis** | |
| --- | --- | --- | --- | --- |
|  | **HR (95% CI)** | **P** | **HR (95% CI)** | **P** |
| **Age (y), ≥72 vs. <72** | 0.92 (0.66-1.28) | 0.619 | - | - |
| **ECOG, ≥2 vs. <0-1** | 2.15 (1.15-4.00) | 0.016 | 1.99 (1.04-3.82) | 0.038 |
| **ISUP group, 5 vs. 1-3** | 1.63 (0.93-2.86) | 0.089 | - | - |
| **ISUP group, 5 vs. 4** | 1.26 (0.83-1.92) | 0.286 | - | - |
| **VM, yes vs. no** | 1.14 (0.69-1.87) | 0.618 | - | - |
| **PSA (ng/ml), ≥12 vs. <12** | 1.30 (0.93-1.82) | 0.125 | - | - |
| **HGB (g/L), <120 vs. ≥120** | 1.42 (1.01-2.01) | 0.046 | 1.37 (0.96-1.94) | 0.083 |
| **ALP (IU/L), ≥160 vs. <160** | 1.56 (1.07-2.26) | 0.021 | 1.23 (0.81-1.85) | 0.333 |
| **LDH (IU/L), ≥220 vs. <220** | 1.60 (1.14-2.24) | 0.006 | 1.43 (1.00-2.04) | 0.048 |
| **NLR (continuous variable)** | 1.07 (1.03-1.11) | 0.001 | 1.07 (1.02-1.11) | 0.002* |
| **dNLR (continuous variable)** | 1.38 (1.18-1.61) | <0.001 | 1.40 (1.20-1.64) | <0.001* |
| **LMR (continuous variable)** | 0.88 (0.80-0.98) | 0.021 | 0.87 (0.79-0.97) | 0.012* |
| **PLR (continuous variable)** | 1.00 (1.00-1.00) | 0.002 | 1.00 (1.00-1.01) | 0.002* |
| **SII (continuous variable)** | 1.00 (1.00-1.00) | <0.001 | 1.00 (1.00-1.00) | <0.001* |
| **SIRI (continuous variable)** | 1.13 (1.06-1.21) | <0.001 | 1.13 (1.05-1.20) | <0.001* |

y = year; mCRPC = metastatic castration-resistant prostate cancer; PSA-PFS = prostate-specific antigen progression-free survival; HR = hazard ratio; CI = confidence interval; ECOG = Eastern Cooperative Oncology Group; ISUP = International Society of Urological Pathology; VM = Visceral metastasis; PSA = prostate-specific antigen; HGB = hemoglobin; ALP = alkaline phosphatase; LDH = lactate dehydrogenase; NLR = neutrophil to lymphocyte ratio; dNLR = derived neutrophil to lymphocyte ratio; LMR = lymphocyte to monocyte ratio; PLR = platelet to lymphocyte ratio; SII = systemic immune inflammation index; SIRI = systemic inflammation response index. *Adjusted for ECOG, HGB, ALP and LDH.
