# Supplementary material for: Analysis of the immune-inflammatory indices for patients with metastatic hormone-sensitive and castration-resistant prostate cancer
Source: BMC Cancer. 2024 Jul 9;24:817. doi: 10.1186/s12885-024-12593-z (PMC11232225; doi:10.1186/s12885-024-12593-z)
Supplement: Supplementary file 10 — Supplementary Material 10. [file 12885_2024_12593_MOESM10_ESM.docx]

**Table S10. Univariate and multivariate analyses of OS in mCRPC cohort.**

|  | **Univariate analysis** | | **Multivariate analysis** | |
| --- | --- | --- | --- | --- |
|  | **HR (95% CI)** | **P** | **HR (95% CI)** | **P** |
| **Age (y), ≥72 vs. <72** | 1.08 (0.72-1.62) | 0.704 | - | - |
| **ECOG, ≥2 vs. <0-1** | 2.27 (1.17-4.38) | 0.015 | 2.72 (1.33-5.56) | 0.006 |
| **ISUP group, 5 vs. 1-3** | 2.86 (1.24-6.62) | 0.014 | 3.79 (1.60-9.01) | 0.002 |
| **ISUP group, 5 vs. 4** | 1.19 (0.72-1.95) | 0.494 | 1.34 (0.80-2.24) | 0.272 |
| **VM, yes vs. no** | 1.74 (0.99-3.03) | 0.053 | - | - |
| **PSA (ng/ml), ≥12 vs. <12** | 1.93 (1.27-2.92) | 0.002 | 1.50 (0.96-2.36) | 0.079 |
| **HGB (g/L), <120 vs. ≥120** | 2.10 (1.39-3.17) | <0.001 | 2.15 (1.39-3.34) | 0.001 |
| **ALP (IU/L), ≥160 vs. <160** | 2.07 (1.31-3.27) | 0.002 | 1.02 (0.58-1.81) | 0.938 |
| **LDH (IU/L), ≥220 vs. <220** | 1.88 (1.24-2.83) | 0.003 | 1.64 (1.03-2.60) | 0.037 |
| **NLR (continuous variable)** | 1.06 (1.03-1.11) | <0.001 | 1.05 (1.01-1.10) | 0.012* |
| **dNLR (continuous variable)** | 1.43 (1.20-1.71) | <0.001 | 1.65 (1.36-1.99) | <0.001* |
| **LMR (continuous variable)** | 0.78 (0.69-0.90) | <0.001 | 0.78 (0.68-0.89) | <0.001* |
| **PLR (continuous variable)** | 1.01 (1.00-1.01) | <0.001 | 1.01 (1.00-1.01) | <0.001* |
| **SII (continuous variable)** | 1.00 (1.00-1.00) | <0.001 | 1.00 (1.00-1.00) | <0.001* |
| **SIRI (continuous variable)** | 1.14 (1.07-1.21) | <0.001 | 1.12 (1.04-1.19) | 0.002* |

y = year; mCRPC = metastatic castration-resistant prostate cancer; OS = overall survival; HR = hazard ratio; CI = confidence interval; ECOG = Eastern Cooperative Oncology Group; ISUP = International Society of Urological Pathology; VM = Visceral metastasis; PSA = prostate-specific antigen; HGB = hemoglobin; ALP = alkaline phosphatase; LDH = lactate dehydrogenase; NLR = neutrophil to lymphocyte ratio; dNLR = derived neutrophil to lymphocyte ratio; LMR = lymphocyte to monocyte ratio; PLR = platelet to lymphocyte ratio; SII = systemic immune inflammation index; SIRI = systemic inflammation response index. *: Adjusted for ECOG, ISUP, PSA, HGB, ALP and LDH.
