# Supplementary figures and images for: Analysis of the immune-inflammatory indices for patients with metastatic hormone-sensitive and castration-resistant prostate cancer
Source: BMC Cancer. 2024 Jul 9;24:817. doi: 10.1186/s12885-024-12593-z (PMC11232225; doi:10.1186/s12885-024-12593-z)

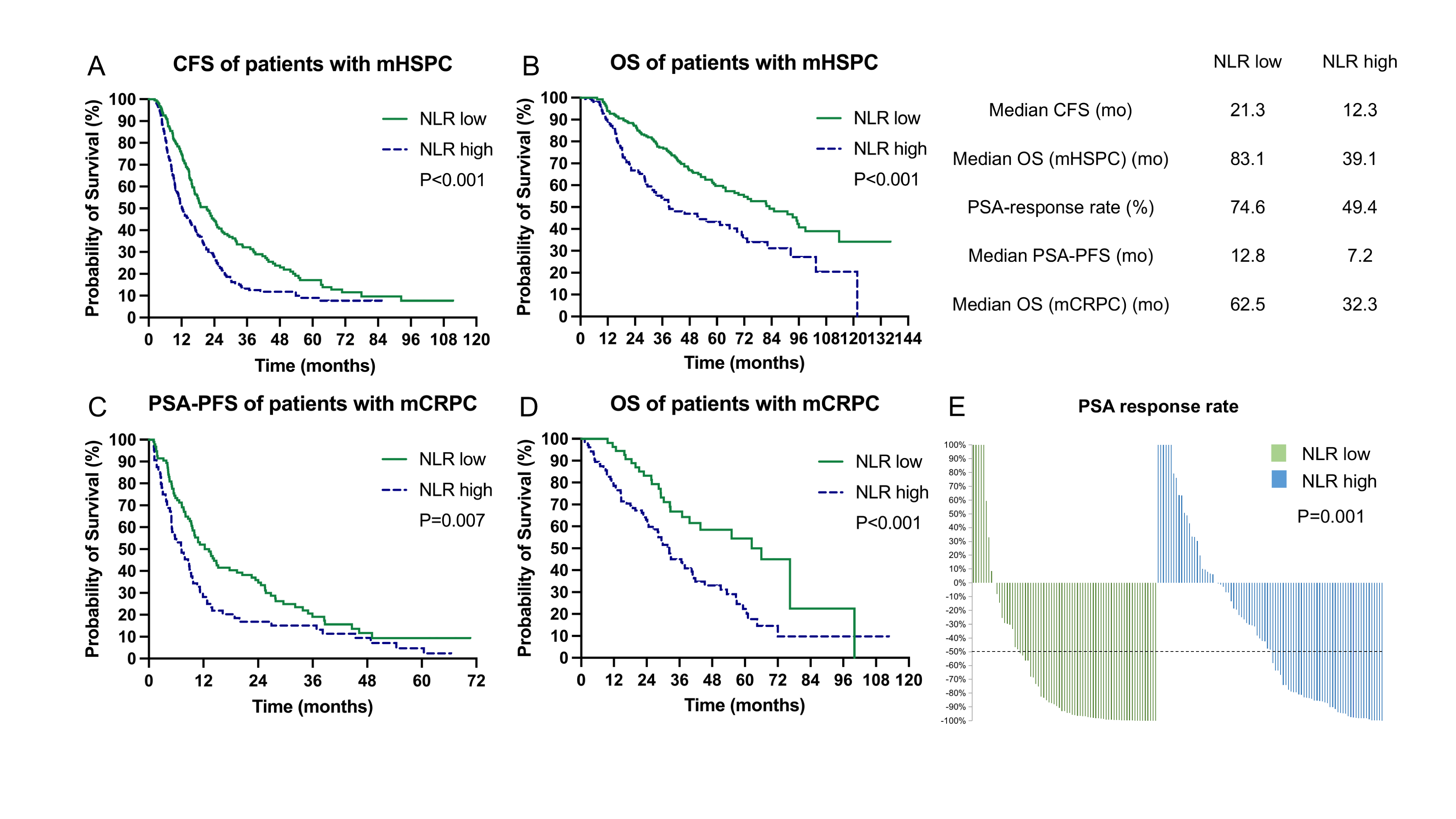

Supplement: Supplementary file 11 — Supplementary Material 11: Fig. S1. The prognostic value of NLR in mHSPC and mCRPC cohorts. (A) Kaplan–Meier curves of CFS for mHSPC cohort; (B) Kaplan–Meier curves of OS for mHSPC cohort; (C) Kaplan–Meier curves of PSA-PFS for mCRPC cohort; (D) Kaplan–Meier curves of OS for mCRPC cohort; (E) PSA response rate for mCRPC cohort. NLR: neutrophil to lymphocyte ratio; mHSPC: metastatic hormone-sensitive prostate cancer; mCRPC: metastatic castration-resistant prostate cancer; CFS: castration-resistant prostate cancer-free survival; OS: overall survival; PSA: prostate-specific antigen; PSA-PFS: prostate-specific antigen progression-free survival. [file 12885_2024_12593_MOESM11_ESM.tif]

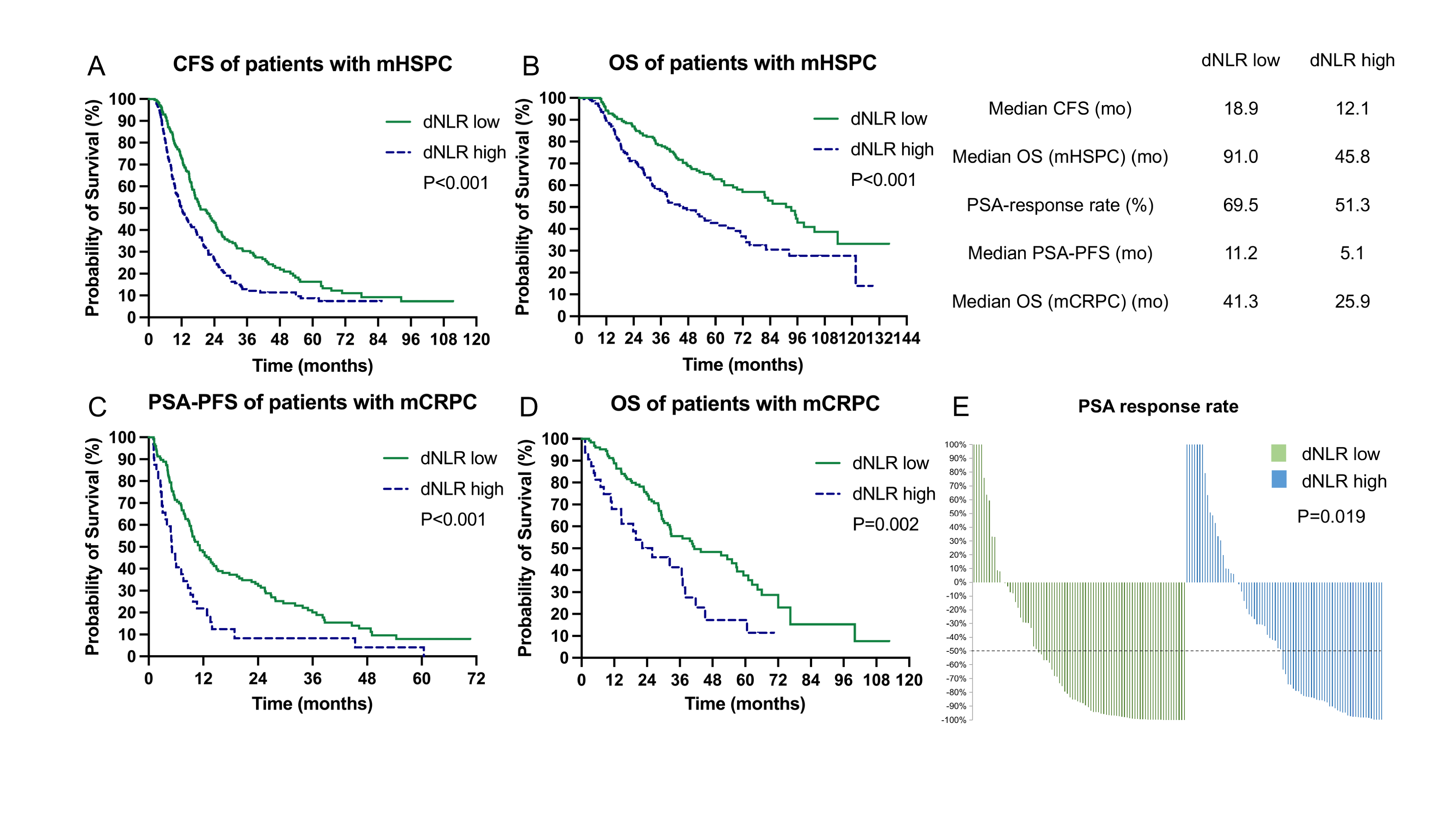

Supplement: Supplementary file 12 — Supplementary Material 12: Fig. S2. The prognostic value of dNLR in mHSPC and mCRPC cohorts. (A) Kaplan–Meier curves of CFS for mHSPC cohort; (B) Kaplan–Meier curves of OS for mHSPC cohort; (C) Kaplan–Meier curves of PSA-PFS for mCRPC cohort; (D) Kaplan–Meier curves of OS for mCRPC cohort; (E) PSA response rate for mCRPC cohort. dNLR: derived neutrophil to lymphocyte ratio; mHSPC: metastatic hormone-sensitive prostate cancer; mCRPC: metastatic castration-resistant prostate cancer; CFS: castration-resistant prostate cancer-free survival; OS: overall survival; PSA: prostate-specific antigen; PSA-PFS: prostate-specific antigen progression-free survival. [file 12885_2024_12593_MOESM12_ESM.tif]

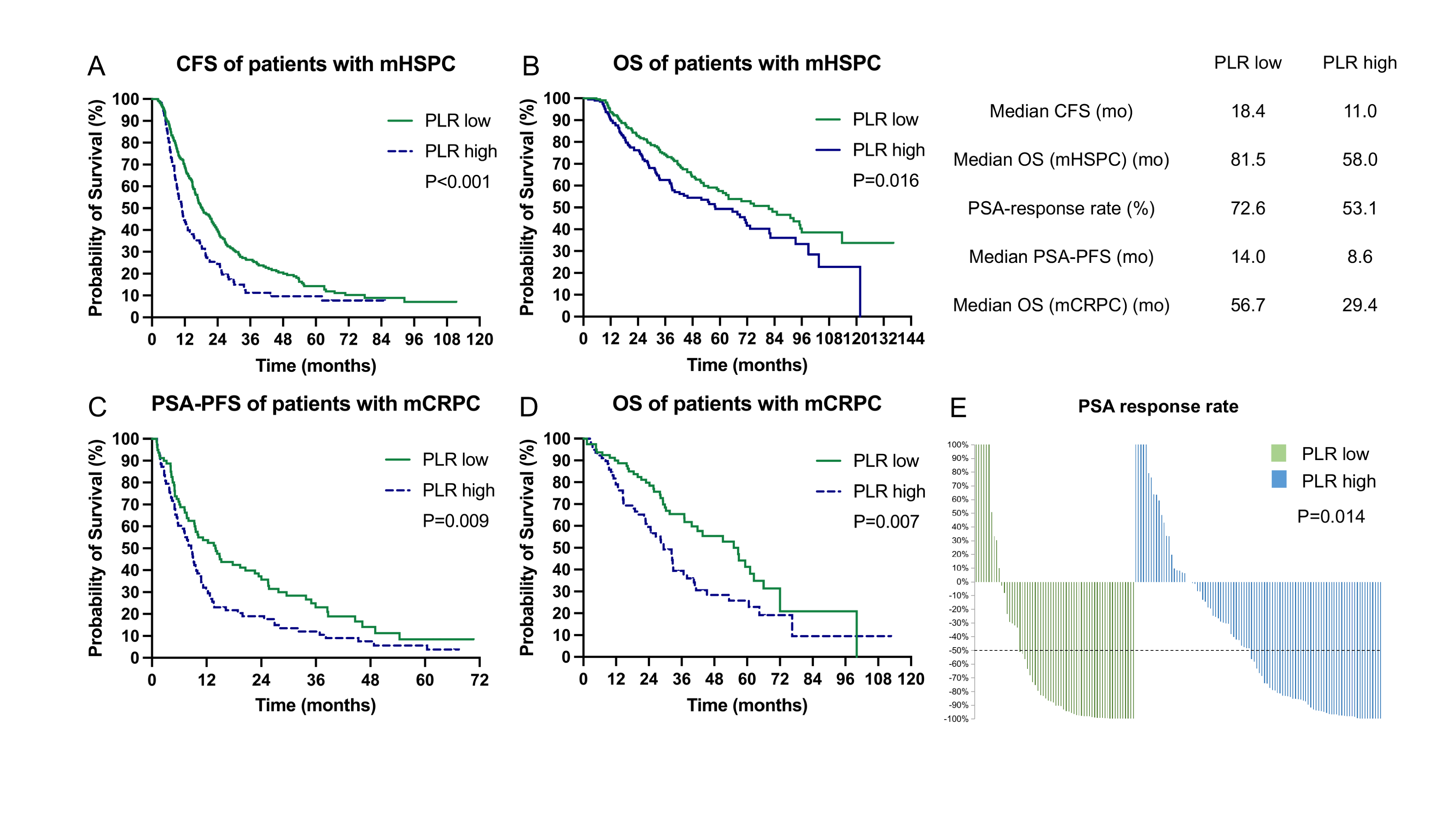

Supplement: Supplementary file 13 — Supplementary Material 13: Fig. S3. The prognostic value of PLR in mHSPC and mCRPC cohorts. (A) Kaplan–Meier curves of CFS for mHSPC cohort; (B) Kaplan–Meier curves of OS for mHSPC cohort; (C) Kaplan–Meier curves of PSA-PFS for mCRPC cohort; (D) Kaplan–Meier curves of OS for mCRPC cohort; (E) PSA response rate for mCRPC cohort. PLR: platelet to lymphocyte ratio; mHSPC: metastatic hormone-sensitive prostate cancer; mCRPC: metastatic castration-resistant prostate cancer; CFS: castration-resistant prostate cancer-free survival; OS: overall survival; PSA: prostate-specific antigen; PSA-PFS: prostate-specific antigen progression-free survival. [file 12885_2024_12593_MOESM13_ESM.tif]

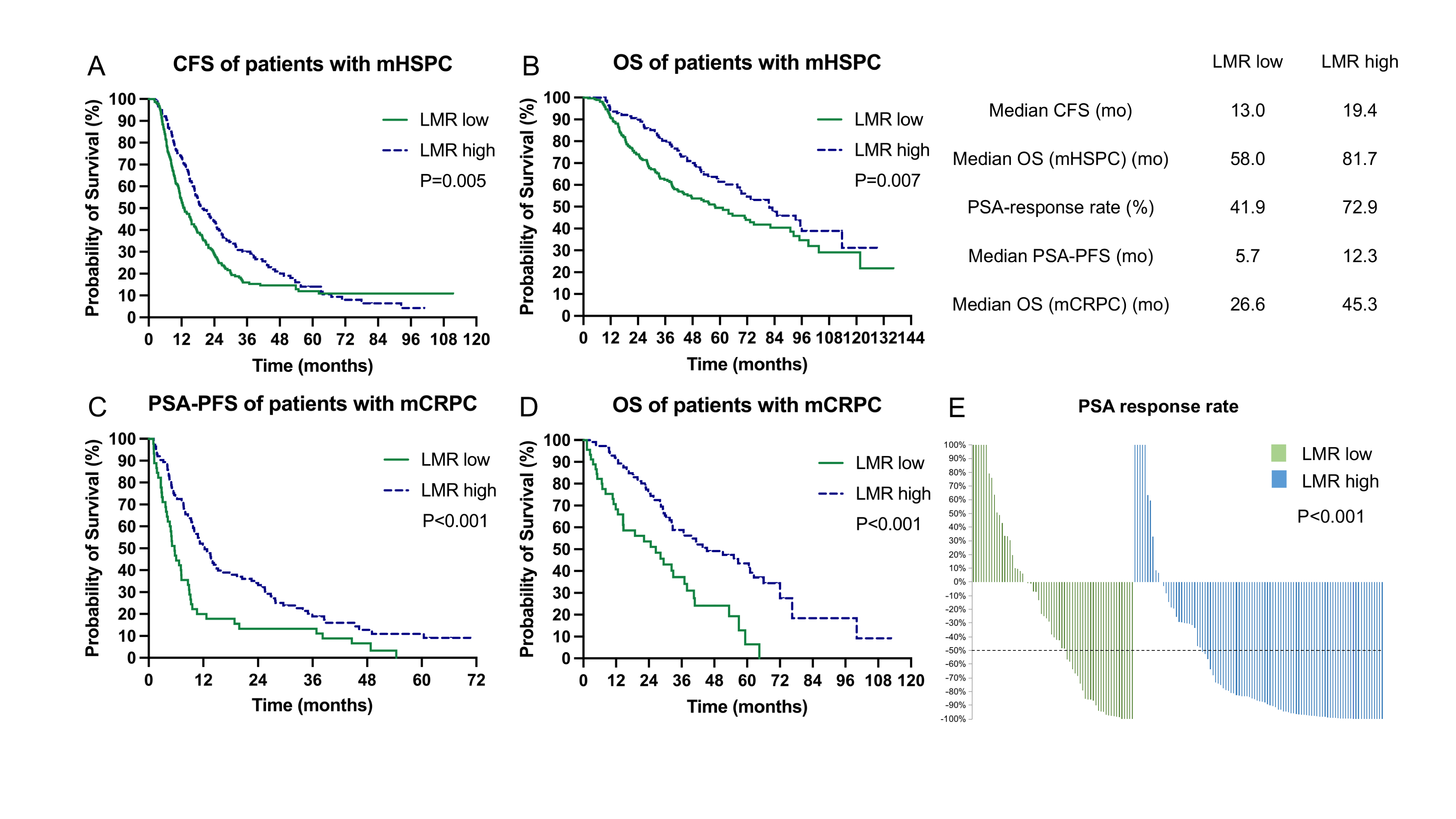

Supplement: Supplementary file 14 — Supplementary Material 14: Fig. S4. The prognostic value of LMR in mHSPC and mCRPC cohorts. (A) Kaplan–Meier curves of CFS for mHSPC cohort; (B) Kaplan–Meier curves of OS for mHSPC cohort; (C) Kaplan–Meier curves of PSA-PFS for mCRPC cohort; (D) Kaplan–Meier curves of OS for mCRPC cohort; (E) PSA response rate for mCRPC cohort. LMR: lymphocyte to monocyte ratio; mHSPC: metastatic hormone-sensitive prostate cancer; mCRPC: metastatic castration-resistant prostate cancer; CFS: castration-resistant prostate cancer-free survival; OS: overall survival; PSA: prostate-specific antigen; PSA-PFS: prostate-specific antigen progression-free survival. [file 12885_2024_12593_MOESM14_ESM.tif]
